# Exploring Domestic Discharge Patterns in Wastewater through LC-HRMS Screening and Temporal Clustering

**DOI:** 10.1021/acs.est.5c02486

**Published:** 2025-07-16

**Authors:** Inga Haalck, Martin Krauss, Werner Brack, Carolin Huber

**Affiliations:** † Department of Exposure Science, 28342 Helmholtz Centre for Environmental Research—UFZ, Permoserstr. 15, 04318 Leipzig, Germany; ‡ Faculty of Biological Science, Goethe University Frankfurt, Theodor-W.-Adorno-Platz 1, 60629 Frankfurt am Main, Germany

**Keywords:** LC-HRMS, non-target screening, wastewater-based epidemiology, k-means temporal clustering, domestic wastewater

## Abstract

Wastewater influent contains valuable epidemiological information, but the complexity of the wastewater matrix poses challenges for data interpretation and linking signals to human exposure. This study aims to analyze daily discharge patterns in influent wastewater to identify recurring patterns for trace organic compounds, particularly those from domestic sources, providing insights into discharge dynamics originating from population chemical consumption and exposure. Over three 24-h periods, hourly composite influent samples from a wastewater treatment plant were analyzed using liquid chromatography coupled to high-resolution mass spectrometry (LC-HRMS). Target and non-target screening revealed over 72,000 features, with 402 target compounds annotated. Temporal k-means clustering of target compounds identified five distinct daily patterns, with two clusters linked to domestic use: one correlated with wastewater flow, representing general daily population activities, and another showing a morning peak, likely associated with morning urine. Based on these patterns, cluster predictions were applied to the non-targeted feature list, prioritizing features with similar temporal trends. This led to 70 additional features associated with the morning peak pattern, with four compounds exemplarily identified. The findings highlight the value of combining targeted and non-targeted analyzes with clustering methods to improve the interpretation of complex wastewater data and unravel chemical discharge patterns linked to population exposure.

## Graphical abstract

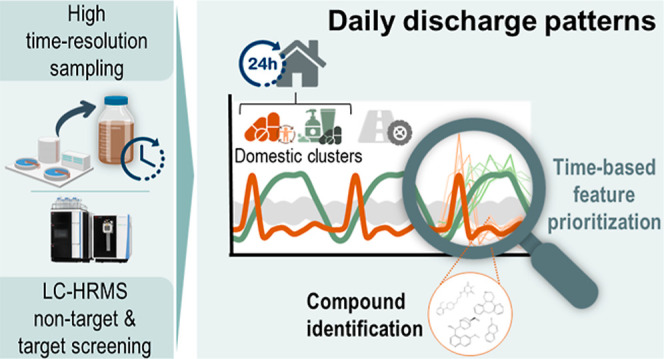

## 1 Introduction

Wastewater influent contains valuable epidemiological information on a community level, which is of interest for new monitoring tools such as wastewater-based epidemiology (WBE) or wastewater-based surveillance (WBS). Here, various information, such as population’s pharmaceutical^1,2^ and illicit drug^3–5^ use, diet,^6^ virus prevalence,^7^ or exposure^8–10^ can be retrieved from wastewater. The approach is based on the analysis of human excretion products (biomarkers),^11^ thus focusing on the urinary and/or fecal fraction of wastewater.

However, the chemical mixture found in influent wastewater is very complex, including not only domestic contributions, such as human excretion products, but also other inputs, such as industrial effluents or surface runoff. This complexity not only increases analytical challenges, but also complicates data interpretation and the link of results to human consumption and exposure.^12,13^ Especially for compounds not intended for human consumption, distinguishing human urinary metabolites from other transformation products formed prior to exposure or in the sewer system is often challenging, but it would be necessary to fully confirm internal exposure and achieve an accurate exposure assessment through WBE.^13^

Pinpointing those compounds in wastewater stemming exclusively from domestic activities (e.g., urination and defecation, showering, among others) may serve as a useful first step in separating WBE-relevant features from other contributing sources. The analysis of daily discharge patterns in influent wastewater may enhance the understanding of chemical source profiles, allowing the identification of the link between chemicals and specific activities. Therefore, we hypothesize that compounds originating from the same source would exhibit the same daily discharge pattern and could be grouped accordingly. Using known chemicals with a known source as a reference, this temporal co-occurrence could then provide valuable indications for chemicals with an unknown source. For instance, evaluating the pattern of compounds known to be excreted by humans (e.g., pharmaceutical metabolites) might provide a strong indication of human internal exposure to other temporally co-occurring compounds.

Temporal trend analysis has already been applied in several studies, such as assessing water contamination dynamics,^14–17^ as early warning system for newly emerging contaminants^18^ or spill detection.^19^ Additionally, evaluating the dynamics of chemicals revealed in several cases valuable information regarding the source and origin of contaminants in surface water^20^ or wastewater.^21,22^

This study aims to analyze daily discharge patterns in influent wastewater to identify recurring patterns for compounds, particularly those from domestic sources, isolating one contributing factor for human exposure and gaining in-depth insights into discharge dynamics. Hourly composite influent samples from a medium-sized wastewater treatment plant (WWTP) were taken for 24 hours on a typical working day, with 3 days as replicates. With targeted and non-targeted LC-HRMS screening combined with clustering methods, we explored discharge patterns of known compounds, and subsequently prioritized non-targeted features exhibiting the same patterns of interest.

## 2 Methods and Materials

### 2.1 Chemicals

The list of target chemicals contained 582 compounds with available reference standards. It covered substances that are typically found in surface water and wastewater such as pharmaceuticals, personal care and household products (PCPs), pesticides, as well as selected transformation products. Additionally, compounds like per- and polyfluoroalkyl substances (PFAS), surfactants, and dyes were grouped together under the category of “other” substances. A full list of all target compounds can be found in the Supporting Information, Tables SX1-2.

### 2.2 Wastewater Sampling

The sampling was performed at the influent of the WWTP in Markkleeberg, Saxony, Germany with a population size of approximately 37,400 inhabitants and 95% of the wastewater contributed from domestic sources. Influent wastewater samples (50 mL) were collected time-proportionally every 5 min for 24 h and composited as hourly samples within the sampling device. The sampling was repeated on three different days. Further details are given in Supporting Information, Section S1 and Tables S1–S2.

### 2.3 Sample Extraction and Chemical Analysis

The sample extraction procedure included filtration, followed by solid phase extraction (SPE) with a Chromabond HR-X (Macherey-Nagel) sorbent and is fully described in Supporting Information, Section S2. A mixture of internal standards (IS) (Supporting Information, Table SX3) was added after reconstitution for quality control of the analysis and data processing to compensate for matrix effect differences among samples and calibration standards for the quantification. The analysis was performed on a Vanquish HPLC system (Thermo Scientific) coupled to a quadrupole-Orbitrap instrument (Exploris 480, Thermo Scientific) operated in positive and negative electrospray ionization mode (ESI+/ESI-) acquiring MS^1^ and MS^2^ information using Top-8 data-dependent (dd) acquisition. Further details on the chromatographic system and the settings of the mass spectrometer can be found in Supporting Information, Section S3 and Table S3.

In every sample batch (1 day), procedure blanks (LC–MS grade water) and two spiked LC-grade water samples at two levels (200 ng/L and 5 μg/L) were extracted and measured within the sequence. Additionally, 24-h composite samples of each day spiked with two levels (200 ng/L and 5 μg/L) were included as quality control (QC). The blanks and QC samples were spiked with the same IS mixture as the samples (Supporting Information, Table SX3). The samples were analyzed in a randomized order and solvent blanks were injected every 12 samples to monitor for potential carryover. The QA/QC samples, along with 12 of the hourly samples were measured twice to assess the analytical reproducibility. The results of the QC can be found in Supporting Information, Section S4 and Figures S1–S3. Before the first sampling campaign, field blanks (*n* = 2) were taken at the WWTP with tap water using the same sampling device.

### 2.4 Data Processing

The Thermo.raw files were converted to the .mzML format using the MSconvert tool of ProteoWizard.^23^ These files were processed in MZmine^24^ (version 3.1.0) to obtain an aligned feature list, including an annotation of potential adducts through ion identity networking. The detailed description of all applied MZmine processing steps and settings can be found in Supporting Information, Table S4.

For compound identification, MS/MS library search was performed in MZmine, including NIST (version 17), MassBank of North America (MoNA), MassBank EU (version 06-24), the Human Metabolome Database (HMDB),^25^ MS-DIAL (version 15) and the aggregated GNPS library^26^ (including partly the aforementioned libraries). Target compounds and IS were annotated with *m*/*z*, retention time (RT) and confirmed with available MS_2_ -spectra. Peak shapes of the targeted compounds were evaluated and retention times were adjusted using the shiny app RtadjusteR.^27^

The final aligned peak list containing *m*/*z*, RT and peak intensity information was exported as .csv file and further processed in R (version 4.3.0). Further cleaning of annotations, such as removing duplicate annotations was accomplished by using the R package MetaboAnnotation (version 1.6.1).^28^ Features with average signal intensities below 1.5-times of the average intensity in the blank samples were removed in an initial filtering step. To further refine the feature list for following data processing, additional filtering steps were applied, which are explained in Section 2.6. Annotations of ESI+ and ESI– modes were merged into a single list, keeping the annotation of the ion mode that showed a higher signal intensity. Semi-quantification was conducted by associating the IS with the closest retention time for each compound. Subsequently, linear regression with 1/*x* -weighting factor was employed to determine the response factors and calculate the concentrations. The R scripts containing all steps for the semi-quantification can be found in the GitHub repository of this project (https://github.com/chufz/WBEpattern).

The structure elucidation of non-targeted features of interest was performed using the graphical interface of SIRIUS (version 5.8.3)^29^ and CANOPUS.^30^ Parameters are found in Supporting Information, Figures S4 and S5. To evaluate the non-targeted features, the proposed structures and CANOPUS compound classes were used, while a threshold of 0.6 for the ClassyFire classes was applied. To assign a confidence level to each annotated compound, we employed the widely recognized Schymanski confidence scale.^31^ This scale categorizes annotations into five confidence levels: Level 1 (confirmed with in-house reference standard), level 2 (spectral library annotation), level 3 (formula and structure identified through SIRIUS), level 4 (formula identified through SIRIUS), and level 5 (unresolved or unknown structure). A full overview on the data processing steps is given in Figure 1.

**Figure 1:**
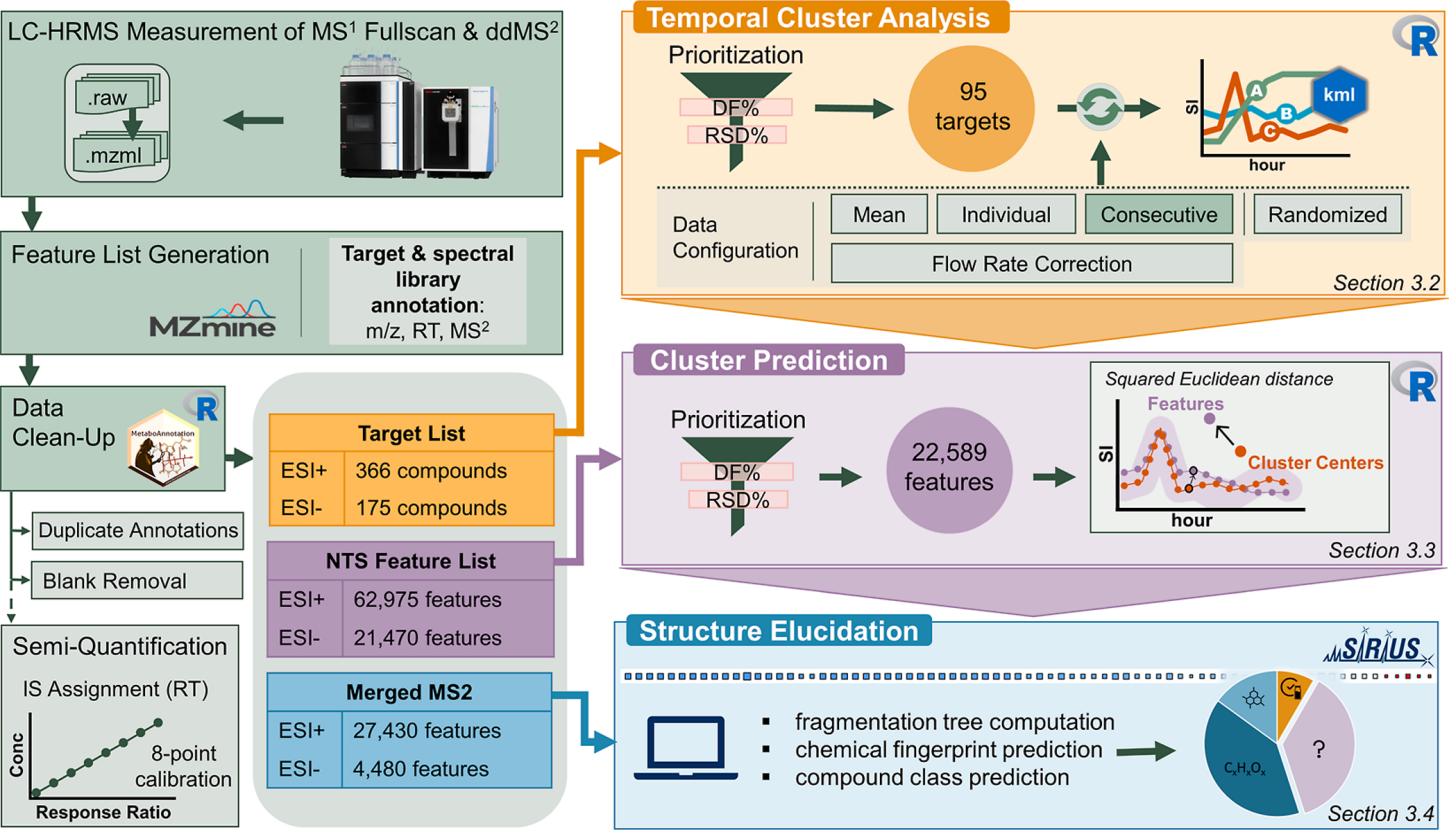
Overview of the data acquisition and analysis, including targeted and non-targeted screening and cluster analysis.

### 2.5 Cluster Analysis

Cluster analysis was performed based on the target annotations described in the previous section. Additionally, clustering was performed on the complete non-target feature list to compare the results with those from the target compounds. Prior to the cluster analysis, the target compounds were prioritized based on their detection frequency (occurrence in >85% of samples) and their variance during the sampling period. Any variation in sample composition - and the resulting matrix effects - or any measurement performance across the hourly samples was expected to contribute no more variability than that observed for the IS. Therefore, only compounds with a variance >45% across all samples, corresponding to 1.5 times the maximum variance observed for the IS (30%), were retained to differentiate between background noise or technical variability and features showing an actual temporal pattern over 24 hours. The remaining non-detects were replaced by one-quarter of the minimal measured value.

Various data configurations were explored as inputs for the cluster analysis, including separating the sampling days, calculating the hourly averages across the three individual sampling days, and treating the data as a single 72-h consecutive data set. Additionally, the influence of flow rate correction on the cluster analysis was evaluated. To confirm that the cluster analysis yields meaningful results only when temporal trends are present, the results were also compared to a randomized sample order.

For all tested data configurations, the peak heights of the targets were log2-transformed and scaled to unit variance in order to ensure that all variables are spread over the same range and have the same variance (eq 1).

1 $$z_{ij}=\frac{x_{ij}-\mu_{i}}{\sigma_{i}}$$

with the variables *z*_*ij*_ as the scaled signal intensity of compound *i* for sample *j*, *x*_*ij*_ the measured signal intensity of compound *i* for sample *j*, μ_*i*_ the mean of compound i across all samples and σ_*i*_ representing the standard deviation of the signal intensities of compound i across all samples.

Cluster analysis was performed in R using the kml package (version 2.4.6.1).^32^ Adaptations to the kml function were based on a previous study,^14^ such as using the diss.CORT function from the TSclust package. The number of clusters was chosen based on the quality criteria provided by the kml package (e.g., Calinski & Harabasz or Ray & Turi criterion) and target compounds were filtered after cluster analysis based on their probability of belonging to the specific cluster (>90%). The cluster assignments of the remaining targets and the centroids of the obtained clusters were exported for further evaluation.

To enhance the visualization of the identified temporal patterns, separate generalized additive models (GAMs) were fitted to each cluster using the mgcv package (version 1.9.1).^33^ All R-scripts used for cluster analysis are available for reproducibility purposes (https://github.com/chufz/WBEpattern).

### 2.6 Cluster Prediction for Non-targeted Features

To predict how the non-targeted features would align with the clusters identified in the preceding cluster analysis on target data, the data was first prepared and filtered using the same procedure as for the target compounds, including filtering based on detection frequency and variance, log2-transformation and scaling. The exported cluster centroids were then used to calculate the squared Euclidean distance to the non-targeted features following eq 2

2 $$D\left(i,j\right)=\sum_{t=1}^{m}{\left(X_{i,t}-C_{j,t}\right)}^{2}$$

with the variable *D*(*i*,*j*) as the squared Euclidean distance between feature *i* and cluster centroid *j*, the value *x*_*i,t*_ of the *i*-th feature at time point *t*, *y*_*i,t*_ as the value of the *j*-th cluster centroid at time point t and *m* as the total number time points (*n* = 72).

After calculating the distances between each feature and cluster centroid, the closest cluster for each feature was identified. To retain only those features with a good fit to a specific cluster, the ratios of the distance between the closest and second closest clusters were calculated and features with a ratio larger than 1.5 were retained. This ensured that features were only assigned to a cluster when the assignment was unambiguous. Additionally, based on the distances of the initial target compounds to their clusters, the calculated median was used as a threshold for the non-targeted features.

## 3 Results and Discussion

### 3.1 Target Annotation and Semi-quantification

Out of 582 target compounds, 402 were detected in at least one wastewater sample, while 131 compounds showed a detection frequency of 100%. A total of 185 compounds were measured with a detection frequency of 85%, which was set as the threshold for the subsequent data processing steps (Supporting Information, Figure S6). Further details on the data evaluation can be found in Supporting Information, Section S7.

The average concentration levels of the semi-quantified compounds in the 24-h composite samples were similar to average ranges already observed in other studies, e.g. for bezafibrate (340 ng/L vs. 9.4–1,471 ng/L,^1,34–36^ ), carbamazepine (499 ng/L vs. 25–1,357 ng/L^1,34–36^ ), furosemide (121 ng/L vs. 116–1,500 ng/L^1,34,35^ ) or mycophenolic acid (1,208 ng/L vs. 1,762–5,392 ng/L^1,35^ ). In some cases, the observed concentrations were higher than those observed in other studies, e.g. for cetirizine (958 ng/L vs. 87–464 ng/L,^1,34,35^ ), possibly due to different sampling seasons.^37^ All concentrations can be found in Supporting Information, Table SX4.

While comparing the concentration levels between 24-h composite samples and 1-h samples, we observed cases of non-detects in the 24-h composite samples for compounds, that were detected up to 17 times in the hourly samples, namely 4-hydroxybenzotriazole, clindamycin, dicyclohexylurea, eprosartan and lincomycin. Additionally, for some compounds, the daily maximum levels were found to be up to 10 times higher than the average levels in the 24-h composite samples, e.g. for 1,3-diphenylguanidine (mean: 276 ng/L vs. daily maximum: 2,744 ng/L), bezafibrate (339 vs. 2,570 ng/L), genistein (200 vs. 2,065 ng/L) and mycophenolic acid (1,208 vs. 8,524 ng/L).

As shown in other studies, temporal high-resolution sampling is beneficial not only to understand concentration dynamics, but also to avoid an underestimation of peak concentration levels from composite sampling.^17,21^ We assume that the lower concentrations and non-detects in the 24-h composite samples compared to the 1-h samples are likely due to the dilution of less-frequently discharged compounds during the 24-h compositing, potentially reducing their overall concentration in the composite sample. Another reason could be increased matrix effects, as stronger ion suppression for the IS signals in the 24-h composite samples has been observed (Supporting Information, Figures S1–S3).

### 3.2 Temporal Clustering of Target Compounds

First, the cluster analysis was tested on both, target compounds and all non-targeted features. The unsupervised clustering of the non-targeted features revealed some difficulties in the interpretation of meaningful patterns due to the absence of a prior relevance-based prioritization of the features in relation to the research question. In addition, compounds with a high potential for adduct or fragment formation or multiple co-occurring congeners (e.g., surfactants) might dominate the clustering result, potentially obscuring more relevant patterns. Thus, clustering of the target compounds, selected to reflect the urban environment, provided a clearer picture.

The range of variation in signal intensities of the IS was 13–30% in ESI+ (*n* = 22) and 23–28% in ESI– (*n* = 3). Therefore, only features with a variation >45% were considered, focusing on temporal trends that exceed potential technical shifts in sensitivity or differences in matrix effects (Supporting Information, Figures S2 and S3). Further evaluation of the total ion chromatogram between the hourly samples supported the assumption that, for this case study, there was no significant shift in matrix composition or matrix effects over the course of the day that could bias the cluster analysis. However, this may differ at different sample sites, where additional corrections (e.g., normalization with IS or postcolumn infusion of a reference) may be necessary.

Different data treatment strategies for clustering of the target compounds were also explored. The evaluation summary and reasoning for choosing to cluster the data set as one 72-h continuous period can be found in Supporting Information, Section S7 and Figures S7–S13.

#### 3.2.1 Observed Temporal Trends

Based on the reasons given in the previous section and Section S7, 95 prioritized target compounds were further analyzed in-depth in the selected cluster analysis. A total of 90 target compounds showed a probability of belonging to their assigned clusters greater than 90%. Figure 2A summarizes the modeled GAMs for each cluster and day, combined with the flow rate measured at the WWTP. The full list of target cluster assignments can be found in Supporting Information, Tables SX1–2. Normalizing signal intensities by flow rate before cluster analysis revealed similar trends to those without normalization but tended to overshadow the temporal patterns of some target compounds (Supporting Information, Figure S11). Hence, we tended for the interpretation without normalization.

**Figure 2:**
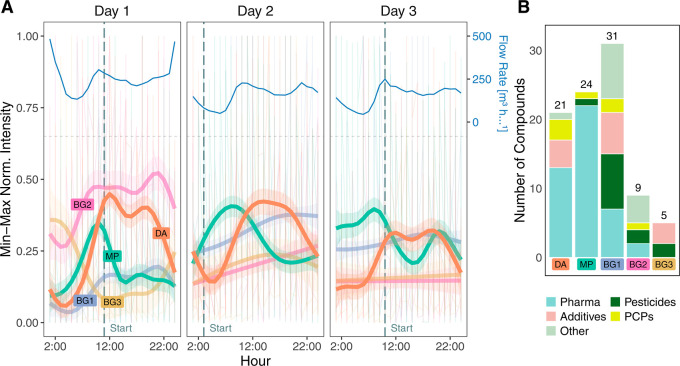
(A) Temporal trends of the identified clusters (bottom) and influent flow rate (top) for three replicate sampling days. The starting point of the sampling is indicated by the gray dashed line. (B) Compound classes of the annotated target compounds in each cluster (DA: daytime activity, MP: morning peak, BG [1–3]: background patterns).

The first cluster (DA = daytime activity) showed a repeated pattern over the three sampled days, with peak intensities between 10 a.m. and 8 p.m. About 23% (*n* = 21) of the targets were assigned to this cluster, including mainly pharmaceuticals, personal care and other household products, such as dioctyl sulfosuccinate or tri-*n*-butyl citrate. These are, among other applications, used in cleaning and washing products.^38^ Tris­(2-butoxyethyl) phosphate, which is used as an antifoaming agent, was also part of this cluster and has already been reported in another study with the highest per capita input among several organophosphorus esters.^39^ Notably, several hypertension medications, including candesartan and telmisartan, were also assigned to this cluster. This observation is consistent with a previous study^21^ that also observed elevated concentrations for these compounds between 8 a.m. and 8 p.m. As seen in Figure 2A, this cluster seems to be influenced by the flow rate of the WWTP, showing a positive Spearman correlation between the cluster center values and the flow rate (ρ = 0.423, Table S5). Based on the target compound range, we interpret this cluster as a representation of the daytime domestic activities of the population, such as showering and elevated use of washing machines or dishwashers. Therefore, this cluster was termed the daytime activities (DA) cluster.

Another cluster (MP = morning peak) showed a recurring pattern with maximum intensities in the morning hours. The highest elevation was observed between 6 a.m. and 9 a.m., so an earlier and narrower peak shape compared to the DA cluster. In addition, a negative Spearman correlation was observed between the values of the cluster centers and the flow rate of the WWTP (ρ = -0.424, Table S5). Therefore, this cluster will be called the *morning peak* (*MP*) cluster. Around 27% (*n* = 24) of the targets were included in this cluster, with pharmaceuticals clearly dominating, in addition to some single entries of PCPs (e.g., climbazole) and one pesticide transformation product (2-(diethylamino)-6-methylpyrimidin-4-one). Among the detected pharmaceuticals, a notably high number of metabolites was found (e.g., acetyl-sulfamethoxazole; phenylethylmalonamide; 10,11-dihydro-10,11-dihydroxycarbamazepine and venlafaxine-*O*-desmethyl). In addition, several pharmaceuticals, which are known to be primarily excreted as parent compound were found in this cluster (e.g., fluconazole, amisulpride and sitagliptin).^40^ In summary, this cluster showed an almost exclusive contribution from pharmaceuticals (see Figure 2B), therefore we assume a relation to internal human exposure. Moreover, with its peak occurring in the morning, we suggest, that this cluster likely represents the first morning void of the population, probably capturing the morning urine.

Unlike the MP and DA clusters, the three remaining clusters (BG1-3 = background 1-3) did not show any distinct peaks during the day. In addition, a higher deviation between the sampling days was observed, with no distinct pattern on the last two sampling days, but with an opposing trend to the flow rate on the first sampling day. One explanation is dilution from a rain event occurring on the first sampling day (documented between 8 p.m. and 4 a.m.). While the flow rate in the WWTP increased during the rain event (see Figure 2A), clusters BG1 and BG2 exhibited a minimum in the same period, followed by increasing intensities throughout the day. This dilution also affected clusters DA and MP, showing lower intensities in the night of the first day as compared to the other nights.

Cluster BG1 included around 34% (*n* = 31) of the targets, covering compounds of diverse applications, such as pharmaceuticals, pesticides and industrial products. Many of the compounds in this cluster have limited to no direct consumer uses, but can be discharged from materials in the indoor and outdoor environment, e.g. tetraglyme, di-(2-ethylhexyl)­amine or di-*n*-butyl phosphate.^38^

Cluster BG2 contained only 10% of the target compounds (*n* = 9), with similarly diverse compound classes as cluster BG1. For instance, compounds associated with food and beverages such as piperine and acesulfame, together with compounds with a variety of discharge sources, such as *N*,*N*-dimethyldodecylamine-*N*-oxide and the two PFAS perfluorosuberic acid and perfluorobutanesulfonic acid were found.

The smallest of the clusters (BG3, 6%, *n* = 5), mirrored the increase in the flow rate during the rain event and thereby showed the opposite trend compared to all other clusters. It included the herbicide isoproturon and the herbicide transformation product terbuthylazine-2-hydroxy, in addition to benzothiazole, 2-benzothiazolesulfonic acid and 1,3-diphenylguanidine. The latter three are known as rubber additives or their degradation products, which are reported as ingredients of car tires and where previously associated with road runoff.^21,41,42^ This suggests that this cluster captured the road and surface runoff, which explains the increase during the rain event.

During the two days without rain, the three clusters BG1–3 showed no particular pattern, suggesting that these compounds were discharged without temporal variation under the dry weather conditions observed in this study. Without any rain event during the sampling, we assume that these compounds would have been filtered out by the variance criterion during data processing. These clusters were therefore termed background (BG) clusters. The influence of rain events on discharge patterns was already evaluated in other studies^41,43,44^ and was not the focus of our study. In the further data evaluation, we will therefore solely concentrate on clusters DA and MP, as these can be related to discharge from domestic activities.

#### 3.2.2 Occurrence of Chemicals Linked to Human Excretion

As hypothesized in the beginning, the temporal co-occurrence of chemicals in wastewater with compounds primarily excreted by humans could provide indications for internal human exposure. Therefore, we further examined the distribution of pharmaceuticals, as they are intended for human consumption and with no pharmaceutical industry influencing the studied WWTP, their occurrence might be most closely related to internal human exposure. As shown in Figure 2B, four out of five clusters included pharmaceuticals, yet in different fractions. We assessed, how the different temporal trends could be related to the therapeutic area, intake frequency and schedule (e.g., morning, night or on demand), with particular emphasis on the elimination half-life and excretion pathway.

The small number of pharmaceuticals contributing to the two background clusters BG1 and BG2 did not relate to any specific therapeutic area. With a few exceptions, nearly all compounds assigned to these clusters were parent compounds of pharmaceuticals that are reported to be predominantly excreted as metabolites.

The DA cluster contained a larger number of cardiovascular drugs, such as hypertension medication (*n* = 4) and lipid-lowering drugs (*n* = 2). Cardiovascular drugs are the most commonly prescribed medications in Germany, with four out of the six above-mentioned compounds ranking among the top 100 in terms of daily prescribed doses in Germany in 2023.^45^ About half of the compounds in this cluster are prescribed once per day, at a time decided by the patient. The other half is taken on demand or up to three times daily. The elimination half-lives differ accordingly (range: 1–80 h), which explains the spread discharge between 10 a.m. and 8 p.m. Eight out of the 13 chemicals contributing to this cluster are reported to be primarily excreted in a metabolized form.

The MP cluster was more specific and included many psychotropic medications such as antidepressants (*n* = 4), antipsychotics (*n* = 4) and antiepileptics (*n* = 3). Most of these compounds are prescribed once per day at a time chosen by the patient, but some compounds are specifically recommended to be taken at night, as they are also used as treatment for insomnia^40^ (*n* = 4). The majority of compounds in this cluster are reported with an elimination half-life of 6 h and longer, enabling an overall more cohesive discharge pattern as compared to the DA cluster. Moreover, the MP cluster had the highest proportion of urinary metabolites (*n* = 5) and parent compounds, which are reported to be the mainly excreted form (*n* = 7). This reinforces the interpretation of its association with morning urine.

Therefore, we hypothesize that non-targeted features associated with the temporal pattern observed for the MP cluster may be more relevant when examining internal human exposure.

### 3.3 Cluster Assignment of Non-targeted Features

In a next step, an assignment of the non-targeted features to the identified clusters was predicted. After filtering based on detection frequency (DF > 85%) and temporal variation (RSD > 45%), the final inclusion list for the cluster prediction contained 22,589 features, including also the annotated 90 target compounds (Supporting Information, Figure S15).

The Euclidean distance of each non-targeted feature to the centroids of the clusters was calculated at each time point (*n* = 72) and summed up to determine the overall closest cluster for each feature. The prioritization described in Section 2.6 reduced the feature list by more than 80% to 3,757 features (Supporting Information, Figure S16). To illustrate the underlying data and the concept of relative distances applied during both cluster analysis and cluster prediction, Figure 3A presents the three closest target compounds for the MP and DA cluster. As all prioritized target compounds were still in the feature list, their correct assignment to their original cluster during the cluster prediction was used as a quality control for the method.

**Figure 3:**
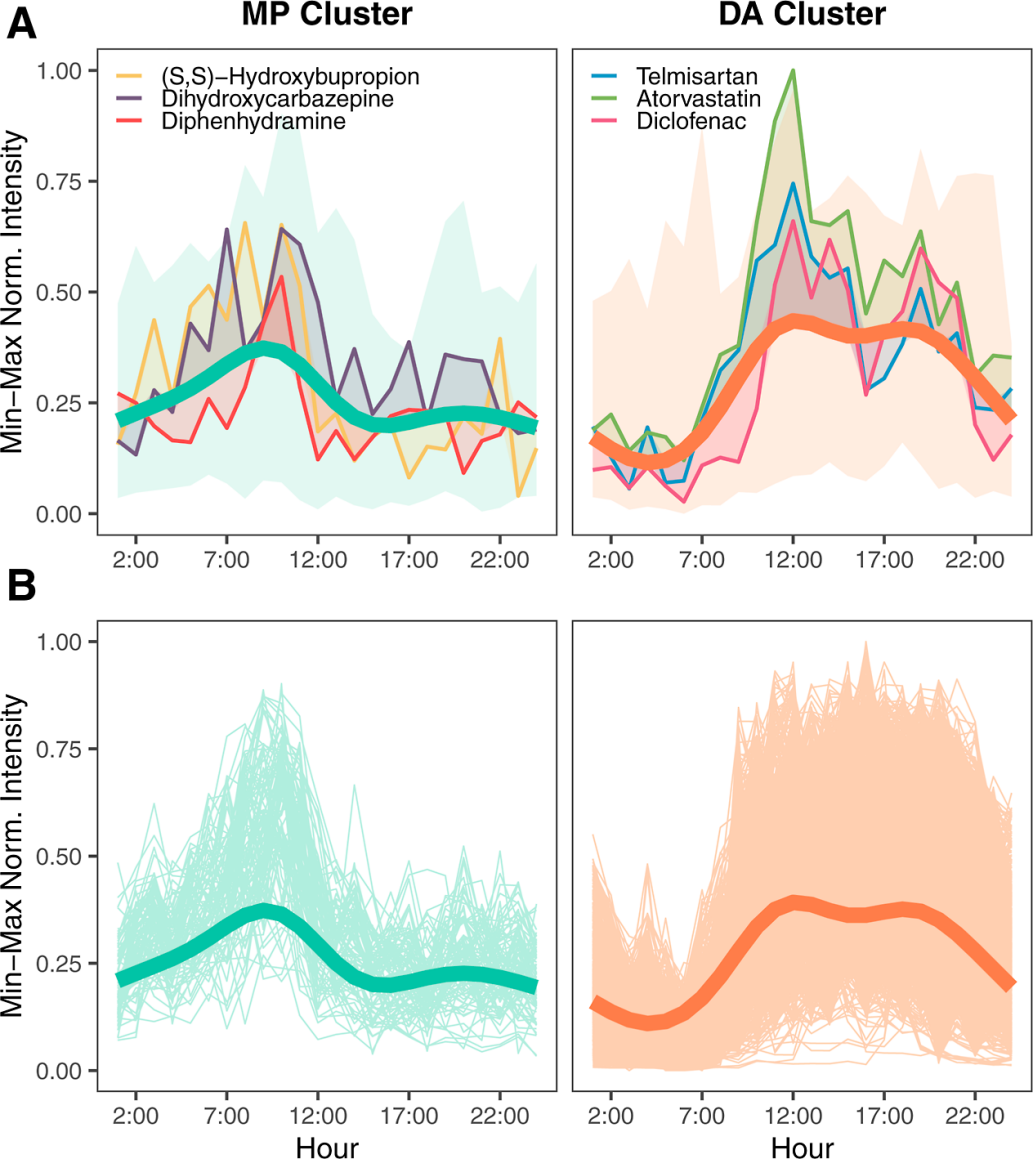
Temporal patterns of to the morning peak (MP) and daytime activities (DA) clusters: (A) The three closest target compounds highlighted in front of the variation of the target compounds. (B) Non-targeted features assigned to each cluster. The thick lines in (A) and (B) illustrate the GAM predictions averaged across three sampling days (see Figure S14 for other clusters).

Excluding the target compounds, a total of 1,133 features were assigned to cluster DA, while 70 features were assigned to the MP cluster. The remaining features were distributed among the background clusters (BG1: 1,868 features, BG2: 585 features, BG3: 11 features).

As seen in Figure 3B, with the use of the Euclidean distance, non-targeted features were successfully prioritized and matched to the previously established daily patterns (further visualization for all other clusters, see Supporting Information, Figure S17).

### 3.4 Characterization of Non-targeted Features

Considering the observed specificity of the MP cluster and its possible association with morning urine, detailed structure elucidation was mainly focused on the MP cluster. Merged dd-MS^2^ data were available for 59 of the 70 non-targeted features assigned to this cluster, which were further analyzed for structure elucidation.

A first classification of all features was conducted based on the ClassyFire hierarchical classification scheme^46^ to compare cluster compositions. While most classes present in the MP cluster were common across all clusters, some, such as cinchona alkaloids (a naturally occurring compound with potential medicinal use) and benzazepines, benzothiazepines and benzodiazepines (used in psychotropic medications), were exclusively present in the MP cluster (Supporting Information, Figure S18).

The subsequent spectral library annotation and fingerprint prediction of the 59 non-targeted features, yielded in a total of 13 conclusive structure annotations (Supporting Information, Table SX5). Out of these candidates, five reference standards were purchased, and four compounds were successfully verified as quinine, its metabolite 3-hydroxyquinine, urapidil, and desmethyl-mirtazapine (Supporting Information, Figures S19–22), corresponding to Schymanski level 1.^31^ In line with the target screening results of the MP cluster, these compounds have pharmaceutical applications, e.g. urapidil for hypertension, which is excreted up to 15% as the parent compound (half-life: 3.3–7.6 h).^40^ Desmethyl-mirtazapine is known as a human metabolite of the annotated antidepressant mirtazapine (excretion rate ∼5%).^47^ Quinine is a natural compound (cinchona alkaloid) used in treatment against muscle cramps and against malaria in tropical countries, or as a bittering agent in drinks (up to 100 mg/L).^48^ It is excreted up to 20% as parent (half-life: 18 h) or as 3-hydroxyquinine.^40^

One feature, which was initially predicted as D617 (verapamil metabolite), appeared to be a structurally similar isomer, as we observed a different retention time. The remaining annotations, for which no reference standards could be purchased, remained as level 2a (*n* = 2) or 3 (*n* = 6). These also included additional potential pharmaceuticals and primarily human metabolites (Supporting Information, Table SX5), which further supports our assumptions. For 25 features, only a conclusive molecular formula annotation could be achieved (level 4), while 22 features remained unresolved (level 5).

To conclude, we prioritized features based on their temporal patterns, enabling a number of features feasible for in-depth structure elucidation, which resulted in the identification of additional compounds of interest. All elucidated compounds are associated with human metabolism, confirming that the MP cluster represents substances found in (morning) urine.

### 3.5 Discussion of Possible Limitations

This study involved exploratory data analysis of 24-h discharge patterns in raw wastewater, using signal intensities over time and only estimating concentration levels for some compounds. The concentration levels reported in this study are semi-quantified, meaning they only allow for a rough approximation of the concentrations compared to dedicated targeted methods, due to structural differences to the IS and other factors, such as automated signal integration. However, this limitation does not affect the validity of the cluster analysis, which focused on patterns in relative signal variations over time, rather than absolute concentrations. The cluster analysis was based on a comprehensive target screening comprising an extensive list of different compounds. The identified clusters may vary depending on the target compounds chosen for the analysis. However, as previously stated, the clustering of the non-targeted peak list confirmed the prevalence of the observed domestic patterns.

The WWTP chosen for this study was medium-sized, with a relatively short travel time from the households to the facility and a high proportion of domestic input. A different WWTP size or larger sewer network might reveal different time patterns and clusters, either by additional (industrial) source or a dilution and dispersion of the domestic patterns due to a wider span of travel times between the households and the WWTP. The short duration of our study (3 days) might be insufficient to be representative of long-term trends and different environmental conditions. Still, our study serves as pilot study to demonstrate the feasibility and usefulness of the approach for wastewater analysis, providing a foundation for future studies to explore other research questions and validate the approach in different contexts. This study aimed to provide an initial proxy for assessing chemicals stemming from domestic sources to deduce population-level (internal or external) exposure. While we have isolated domestic use as one contributing factor for exposure, we are aware that this reveals only a piece of evidence and further refinement is needed to focus exclusively on internal exposure.

### 3.6 Implications and Future Applications

Wastewater is a valuable source of epidemiological information; however, its complex matrix exacerbates extracting meaningful results and the link to population exposure. By studying the daily discharge patterns, this study offers insights into chemical discharge profiles of different sources (e.g., domestic vs. road runoff), making this approach valuable for chemical source identification. Additionally, the results demonstrate the advantages of high-time resolution sampling, as by avoiding dilution, we could find more compounds and higher maximum concentrations in the hourly samples compared to the 24-h composite samples.

We demonstrate the presence of reproducible and recurring daily patterns, pinpointing two patterns associated with domestic use: one representing general daytime activities and another with a closer connection to human metabolism. The majority of compounds in the latter cluster are associated with pharmaceutical use, which facilitated the assessment of whether these compounds had passed through the human body, enabling the association to (morning) urine. We propose this discrimination as a beneficial tool for monitoring approaches such as WBE, providing additional evidence about the origin of a potential biomarker and supporting the interpretation of other compounds related to human metabolism (e.g., endogenous compounds used for population normalization or to confirm the presence of human excretion), and making conclusions more meaningful. However, in this pilot study, only pharmaceuticals represent chemicals intended for consumption, while WBE is currently seeking to include a wider range of chemicals related to unintentional exposure.^49^ To include them, comparing the temporal patterns and cluster assignments of parent compounds and their human-specific metabolites could provide a more precise understanding of their internal exposure profile, helping to confirm that their source is exclusively related to human exposure.^50^

## Supplementary Material





## Notes

Information on wastewater sampling, sample extraction, chemical analysis, quality control, data treatment and structure elucidation; exclusion criteria for target and non-target screening; comparison of results from different data inputs for cluster analysis; output of generalized additive models; cluster prediction results for non-targeted features; comparison of ClassyFire classes among clusters; MS2 spectra comparison of identified compounds (PDF)
Target list and results of target screening; internal standards; results of the semi-quantification; structure elucidation results (XLSX)
