# Supplementary material for: Exploring Domestic Discharge Patterns in Wastewater through LC-HRMS Screening and Temporal Clustering
Source: Environ Sci Technol. 2025 Jul 16;59(29):15375–84. doi: 10.1021/acs.est.5c02486 (PMC12312085; doi:10.1021/acs.est.5c02486)
Supplement: Supplementary file 1 [file es5c02486_si_001.pdf]

Exploring Domestic Discharge Patterns in Wastewater through LC-HRMS Screening and Temporal Clustering

Supporting Information – Part I

Inga Haalck[1,2], Martin Krauss[1], Werner Brack[1,2] & Carolin Huber[1]*

[1] Helmholtz Centre for Environmental Research – UFZ, Department of Exposure Science, Permoserstr. 15, 04318 Leipzig, Germany.

[2] Goethe University Frankfurt, Faculty of Biological Science, Theodor-W.-Adorno-Platz 1, 60629 Frankfurt am Main, Germany

Corresponding author:

Inga Haalck (email: inga.haalck@ufz.de)

Summary: 28 pages / 8 sections / 5 tables / 22 figures

Table of Contents

Sections

**Section S1:** Wastewater sampling	S4
**Section S2:** Sample extraction procedure	S5
**Section S3:** Further details of the chemical analysis	S6
**Section S4:** Quality assurance and control	S8
**Section S5:** MzMine parameters	S11
**Section S6:** SIRIUS parameters	S13
**Section S7:** Data pretreatment and cluster analysis results	S14
**Section S8:** Non-targeted feature prioritization	S23

Tables

**Table S1.** Start/End times of the sampling campaign.	S4
**Table S2.** Overview of the measured influent flow rate (m^3/h) at the WWTP	S5
**Table S3.** Mass spectrometric parameters for the measurement on a Thermo Fisher Exploris 480.	S7
**Table S4.** MzMine steps and applied parameter settings.	S11
**Table S5.** Spearman rank correlation coefficients of all 5 clusters against the wastewater flowrate.	S21

Figures

**Figure S1.** Total ion chromatogram for ESI+ and ESI- of one exemplary batch day.	S9
**Figure S2.** QA/QC (ESI+).	S10
**Figure S3.** QA/QC (ESI-).	S10
**Figure S4.** SIRIUS parameters – ESI+.	S13
**Figure S5.** SIRIUS parameters – ESI-.	S14
**Figure S6**. Cut-off value applied for detection frequency and relative standard deviation of target compounds in wastewater samples.	S15
**Figure S7.** Quality criteria & cluster pattern when clustering each sampling day individually.	S16
**Figure S8.** Quality criteria & cluster pattern when clustering the data as average of each data point over the day for all three sampling days.	S17
**Figure S9.** Quality criteria & cluster patterns when clustering the data as a consecutive data set.	S18
**Figure S10.** Variability of signal intensities between sampling days.	S19
**Figure S11.** Quality criteria & cluster patterns with flow rate correction, using the data as a consecutive data set.	S19
**Figure S12.** Controlling the observed clustering as non-artefacts by randomized ordering of the sample days.	S20
**Figure S13.** Quality criteria & cluster patterns for a clustering of the non-targeted feature list.	S20
**Figure S14.** Generalized Additive Model (GAM) calculated for the 5 clusters per day.	S21
**Figure S15.** Cut-off values applied for detection frequency and relative standard deviation on the selection of non-targeted features over the sampling period.	S23

Figure S16. Cut-off values for the non-targeted features based on distance ratio and distance threshold. S24
Figure S17. Assigned non-targeted features for each cluster over 72 hours and averaged over 24 hours. S25
Figure S18. ClassyFire Classes annotated in the non-target features of the morning peak cluster compared over all other clusters. S26
Figure S19. Spectra comparison of the measured spectra in wastewater and the reference spectra for quinine. S26
Figure S20. Spectra comparison of the measured spectra in wastewater and the reference spectra for 3-hydroxyquinine. S27
Figure S21. Spectra comparison of the measured spectra in wastewater and the reference spectra for normirtazapine. S27
Figure S22. Spectra comparison of the measured spectra in wastewater and the reference spectra for urapidil. S28

Section S1: Wastewater sampling

The sampling was performed at the influent of the WWTP in Markkleeberg, Saxony, Germany (51.33°N, 12.37°E). The average hydraulic residence time within the connected sewer system is approx. three hours. A total of three sampling campaigns were conducted between April and July 2023. On all three occasions, the sampling was performed on a Wednesday, yet the exact starting point of the 24-hour sampling campaign ranged between 4 am and 11 am. This variation was designed to ensure no potential trends at the beginning or end of the sampling period are overlooked. On two sample days, namely days 2 and 3, no precipitation was reported, while on day 1 light rain was documented between 8 pm and 4 am.

For sampling, a programmable TP5 device (MAXX Mess-u. Probenahmetechnik GmbH, Rangendingen, Germany) was used, which allowed the collection of 24 individual, hourly samples in glass bottles. A volume of 50 mL of influent wastewater was taken every 5 minutes for 24 hours and composited as 1-hour samples, resulting in 72 samples for the three sampling days. For quality control, a fraction (V = 10 mL) of each 1-hour sample was composited into one 24-hour composite sample for each sampling day. The samples were stored at 5°C during the sampling campaign and transport and were frozen at -20°C until extraction. All parameters concerning the sampling (start/end time, wastewater flow rate) can be found in Tables S1-2.

Table S1. *Start/End times of the sampling campaign.*

Sampling Day	Start		End	
	Date	Time	Date	Time
1	19.04.2023	11:00 AM	20.04.2023	10:55 AM
2	14.06.2023	03:00 AM	15.06.2023	02:55 AM
3	05.07.2023	10:00 AM	06.07.2023	9:55 AM

Table S2. *Overview of the measured influent flow rate (m^3/h) at the WWTP*

Hour	Day 1	Day 2	Day 3
01:00	483*	149	142
02:00	349	110	110
03:00	264	82	84
04:00	163*	66	67
05:00	139	59	52
06:00	133	50	45
07:00	151	67	61
08:00	195	127	119
09:00	267	203	214
10:00	304	227	250
11:00	284	226	209
12:00	270	209	207
13:00	250	197	189
14:00	243	193	175
15:00	243	177	182
16:00	218	158	165
17:00	211	158	155
18:00	221	157	162
19:00	234	168	162
20:00	255*	194	181
21:00	260	229	196
22:00	270	206	192
23:00	281	199	193
00:00	468	172	169

* rain documented at that hour

Section S2: Sample extraction procedure

The extraction method was adapted with some modifications from a similar screening application[1]. The wastewater samples were filtered through Whatmann GF/F microfiber filters (0.55 µm, 135 mm diameter) using a vacuum pump. Solid-phase-extraction (SPE) was performed with the SPE-03 device (Promochrom, Richmond, BC, Canada). Chromabond HR-X cartridges (*85 µm, 200 mg, Machery-Nagel. Düren, Germany*) were conditioned with each 1 mL of ethyl acetate and methanol, followed by 2 mL of LC-MS-grade water (*Honeywell*). A volume of 50 mL of sample were loaded on the cartridge and washed with 4 mL of LC-MS grade water. The cartridges were then dried for 30 min under a gentle nitrogen stream. The samples were eluted using each 2 mL of each of ethyl acetate and methanol. The eluent was evaporated under a gentle nitrogen stream until dryness and reconstituted in 500 µL of methanol.

For the analysis, 100 µL of the reconstituted sample was added to 96 µL of LC-grade water and spiked with 4 µL of an internal standard mixture (25 isotope-labeled compounds, listed in SX3).

Section S3: Further details of the chemical analysis

A reverses phase column (2.1 mm × 100 mm, 1.7 µm particle size, Waters BEH C18) was used in both cases and maintained at 50°C with a flow rate of 0.3 mL/min. For ESI+, a gradient elution of water/methanol (with 0.1% formic acid and 2 mM ammonium formate) was employed, starting at 100/0 at 0 minutes, shifting to 0/100 at 15 minutes, maintaining at 0/100 until 21 minutes, returning to 100/0 at 22 minutes, and concluding at 100/0 at 30 minutes. For ESI-, a gradient elution of water and water/methanol (90:10 v/v), both containing 10 mM ammonium bicarbonate, was employed, following the same timing program as described. The injection volume was 5 µL.

HRMS analysis was conducted in both positive and negative electrospray ionization modes (ESI+/ESI-). The method was previously published in Huber et al. 2024[2]. The nominal resolving power was R = 120,000 at m/z 200 for full-scan MS^1 acquisition (scan range 80-1000). Each MS^1 scan event was followed by 8 data-dependent-MS^2 events with an HCD collision energy of 50% and a resolution of 30,000 (scan range 50-500), employing an apex trigger and an exclusion list of known background contamination. Further details are found in Table S3.

Table S3. *Mass spectrometric parameters for the measurement on a Thermo Fisher Exploris 480.*

ESI+	ESI-
**Method Settings**	
Application Mode: Small Molecule<br>Method Duration (min): 30	Application Mode: Small Molecule<br>Method Duration (min): 30
**Global Parameters**	
**Ion Source**<br>Ion Source Type: H-ESI<br>Spray Voltage: Static<br>Positive Ion (V): 2500<br>Negative Ion (V): 2000<br>Gas Mode: Static<br>Sheath Gas (Arb): 45<br>Aux Gas (Arb): 7<br>Sweep Gas (Arb): 0<br>Ion Transfer Tube Temp (°C): 325<br>Vaporizer Temp (°C): 350<br>APPI Lamp: Not in Use<br>Use Ion Source Settings from Tune: False<br>FAIMS Mode: Not Installed<br><br>**MS Global Settings**<br>Infusion Mode: Liquid Chromatography<br>Expected LC Peak Width (s): 20<br>Advanced Peak Determination: False<br>Mild Trapping: False<br>Default Charge State: 1<br>Enable Xcalibur AcquireX method modifications: False<br>Internal Mass Calibration: EASY-IC™<br>Mode: Run Start	**Ion Source**<br>Ion Source Type: H-ESI<br>Spray Voltage: Static<br>Positive Ion (V): 2500<br>Negative Ion (V): 2000<br>Gas Mode: Static<br>Sheath Gas (Arb): 40<br>Aux Gas (Arb): 7<br>Sweep Gas (Arb): 1.5<br>Ion Transfer Tube Temp (°C): 325<br>Vaporizer Temp (°C): 350<br>APPI Lamp: Not in Use<br>Use Ion Source Settings from Tune: False<br>FAIMS Mode: Not Installed<br><br>**MS Global Settings**<br>Infusion Mode: Liquid Chromatography<br>Expected LC Peak Width (s): 20<br>Advanced Peak Determination: False<br>Mild Trapping: False<br>Default Charge State: 1<br>Enable Xcalibur AcquireX method modifications: False<br>Internal Mass Calibration: EASY-IC™<br>Mode: Run Start
**Experiment #1 [MS]**	
Start Time (min): 1<br>End Time (min): 21<br><br>**<u>Master Scan:</u>**<br>**Full Scan**<br>Orbitrap Resolution: 120000<br>Scan Range (m/z): 80-1000<br>RF Lens (%): 50<br>AGC Target: Standard<br>Maximum Injection Time Mode: Auto<br>Microscans: 1<br>Data Type: Centroid<br>Polarity: Positive<br>Source Fragmentation: Disabled<br>Scan Description:<br><br>**<u>Filters:</u>**<br>**Dynamic Exclusion**<br>Dynamic Exclusion Mode: Custom<br>Exclude after n times: 1<br>Exclusion duration (s): 45<br>Mass Tolerance: ppm<br>Low: 8<br>High: 8<br>Exclude isotopes: True<br>Perform dependent scan on single charge state per precursor only: True	Start Time (min): 1<br>End Time (min): 21<br><br>**<u>Master Scan:</u>**<br>**Full Scan**<br>Orbitrap Resolution: 120000<br>Scan Range (m/z): 80-1000<br>RF Lens (%): 50<br>AGC Target: Standard<br>Maximum Injection Time Mode: Auto<br>Microscans: 1<br>Data Type: Centroid<br>Polarity: Negative<br>Source Fragmentation: Disabled<br>Scan Description:<br><br>**<u>Filters:</u>**<br>**Dynamic Exclusion**<br>Dynamic Exclusion Mode: Custom<br>Exclude after n times: 1<br>Exclusion duration (s): 45<br>Mass Tolerance: ppm<br>Low: 8<br>High: 8<br>Exclude isotopes: True<br>Perform dependent scan on single charge state per precursor only: True

**Targeted Mass Exclusion**<br>**Mass List**<br>Mass List Type: m/z<br>Time Mode: Start/End Time<br>Include Intensity Threshold: True<br>Exclusion mass width: ppm<br>Low: 8<br>High: 8<br><br>**Apex Detection**<br>Desired Apex Window (%): 30<br><br>**Data Dependent**<br><br>Data Dependent Mode: Number of Scans<br>Number of Dependent Scans: 8<br><br>**Scan Event Type 1:**<br>**Scan:**<br>**ddMS²**<br>Multiplex Ions: False<br>Isolation Window (m/z): 0.7<br>Isolation Offset: Off<br>Collision Energy Type: Normalized<br>HCD Collision Energy (%): 50<br>Orbitrap Resolution: 30000<br>Scan Range Mode: Define m/z Range<br>Scan Range (m/z): 50-500<br>AGC Target: Standard<br>Maximum Injection Time Mode: Auto<br>Microscans: 1<br>Data Type: Centroid	**Targeted Mass Exclusion**<br>**Mass List**<br>Mass List Type: m/z<br>Time Mode: Start/End Time<br>Include Intensity Threshold: True<br>Exclusion mass width: ppm<br>Low: 8<br>High: 8<br><br>**Apex Detection**<br>Desired Apex Window (%): 30<br><br>**Data Dependent**<br><br>Data Dependent Mode: Number of Scans<br>Number of Dependent Scans: 8<br><br>**Scan Event Type 1:**<br>**Scan:**<br>**ddMS²**<br>Multiplex Ions: False<br>Isolation Window (m/z): 0.7<br>Isolation Offset: Off<br>Collision Energy Type: Normalized<br>HCD Collision Energy (%): 50<br>Orbitrap Resolution: 30000<br>Scan Range Mode: Define m/z Range<br>Scan Range (m/z): 100-600<br>AGC Target: Standard<br>Maximum Injection Time Mode: Auto<br>Microscans: 1<br>Data Type: Centroid

Section S4: Quality assurance and control

The evaluation of the mass spectrometer's performance included an analysis of batch effects, with the stability of RT, m/z, and signal intensity (SI) presented in Figures S1-3. The absolute RT shift was more stable in ESI+ with a standard deviation of 0.01 min, compared to 0.03 min in ESI-. The signal intensities of the internal standards ranged from 13-30% in ESI+ (n = 22) and from 23-28 % in ESI- (n=3).

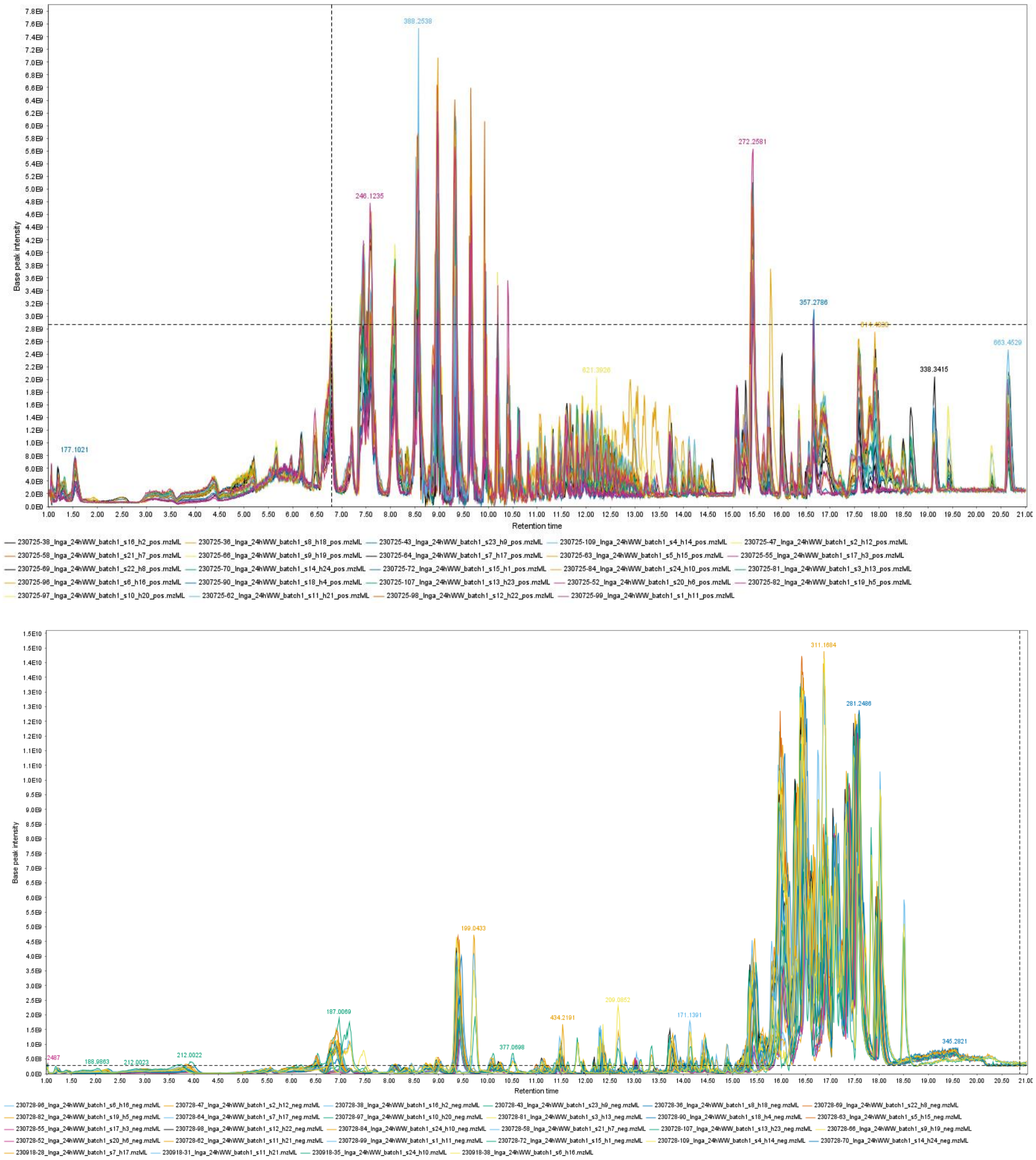

Figure S1. *Total ion chromatogram for (A) ESI+ and (B) ESI- of one exemplary batch day. Here, only minor differences in the overall sample composition between the hourly samples could be observed.*

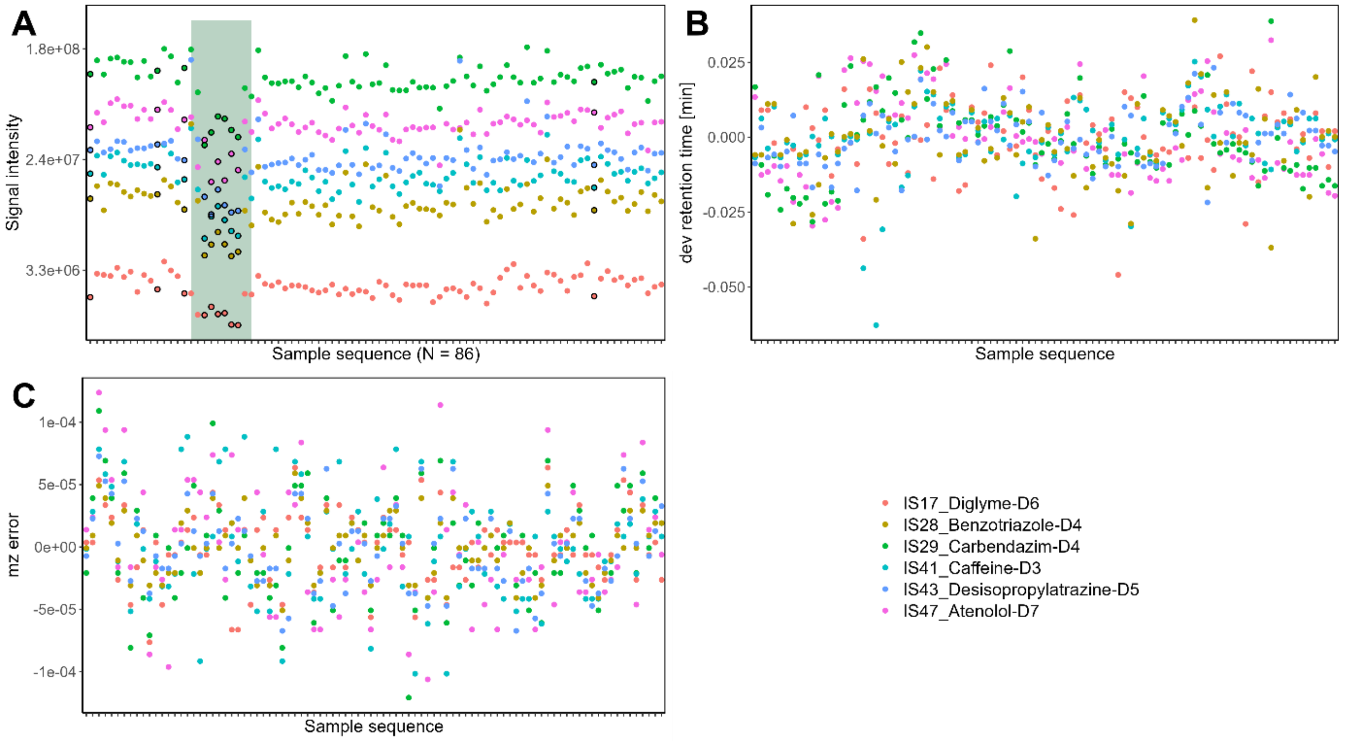

Figure S2. *QA/QC (ESI+): Signal intensity variability (A), retention time shifts (B) and m/z error (C) exemplary displayed for 6 internal standards. The green area in (A) shows the increased ion suppression in the 24-hour composite samples (framed in black).*

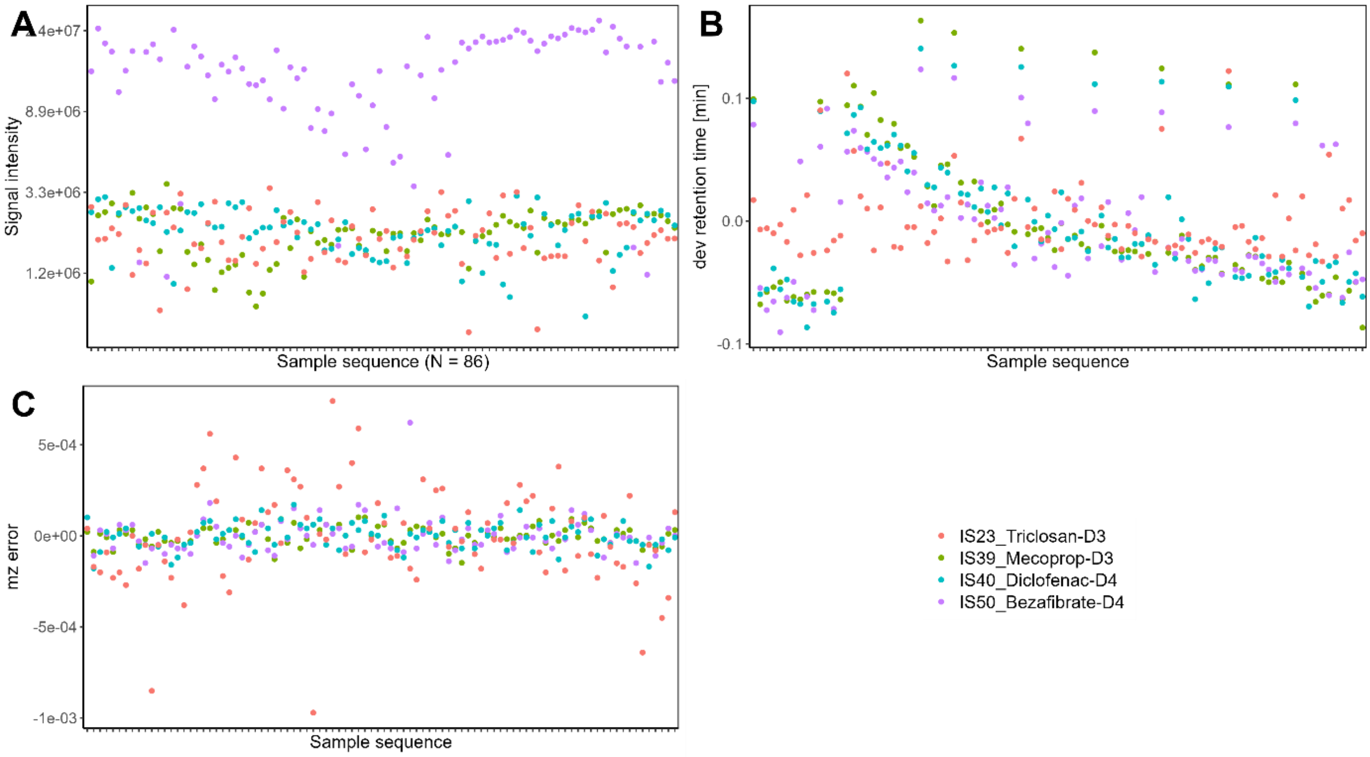

Figure S3. *QA/QC (ESI-): Signal intensity variability (A), retention time shifts (B) and m/z error (C) exemplary displayed for 4 internal standards.*

A total of 62,975 features were detected in positive and of 21,470 features in negative mode. For a total of 44% of features in ESI+, merged dd-MS^2 information was available. In ESI-, this was the case for 21% of the features. After blank removal 56,375 features in positive and 15,973 features in negative mode remained in the list, which were then combined into one list with 72,348 features.

A total of 40 compounds were considered semi-quantifiable, based on the following criteria for quantification: a calibration curve achieving $R^2 > 0.7$, a detection rate within the calibration range higher than 80%, and a recovery rate between 25% and 250% for the sample preparation.

Section S5: MzMine parameters

Table S4. *MzMine steps and applied parameter settings.*

**1**	**Mass detection**
**2**	**ADAP chromatogram deconvolution (Myers et al., 2017)**<br>- Minimum number of scans: 4<br>- Minimum intensity for consecutive scans 5.0E4<br>- Minimum absolute height 1.0E5<br>- m/z tolerance (scan-to-scan): 0.002 m/z or 10 ppm
**3**	**Smoothing (Savitzky Golay algorithm)**
**4**	**Local minimum feature resolver**<br>- Chromatographic threshold: 90%<br>- Minimum search range RT: 0.1<br>- Minimum absolute height: 1.0E5<br>- Min ratio peak top/edge 1.80<br>- Minimum scans: 4
**5**	**13C isotope filter**<br>- m/z tolerance (intra-sample): 0.0015 m/z or 3 ppm<br>- RT tolerance: 0.08<br>- Maximum charge 2<br>- Representative isotope: most intense
**6**	**Isotopic peaks finder:**<br>- m/z tolerance (feature-to-scan): 0.0015 m/z or 3 ppm<br>- maximum charge of isotope m/z: 1

**7**	**Join aligner:**<br>- m/z tolerance (sample-to-sample): 0.0015 m/z or 5 ppm<br>- weight for m/z: 3<br>- retention time tolerance: 0.4<br>- weight for RT: 1<br>- Mobility weight: 1
**8**	**Feature list rows filter**<br>- Minimum aligned features (samples): max of 5 samples or 5%
**9**	**Peak finder (multithreaded)**<br>- Intensity tolerance 20.0%<br>- m/z tolerance (sample-to-sample): 0.002 m/z or 10 ppm<br>- Retention time tolerance: 0.4<br>- Minimum scans (data points): 1
**10**	**Duplicate peak filter**<br>- m/z tolerance: 0.0008 m/z or 1.5 ppm<br>- RT tolerance: 0.070
**11**	**Group MS2 scans with features**<br>- MS1 to MS2 precursor tolerance (m/z): 0.008 m/z or 15 ppm<br>- Minimum relative feature height: 25%<br>- Minimum required signals: 1<br>- Minimum signal intensity (relative, TIMS: 1%
**12**	**Correlation Grouping (metaCorrelate):**<br>- RT tolerance: 0.11<br>- Minimum feature height: 1.0E4<br>- Intensity threshold for correlation: 5.0E4
**13**	**Ion identity networking**<br>- m/z tolerance (intra-sample): 0.0015 m/z or 3 ppm<br>- Check: all features<br>- Min height: 0.0E0
**14**	**Custom database search (target list)**<br>- m/z tolerance: 0.001 m/z or 5 ppm<br>- RT tolerance: 0.2<br>- Mobility time tolerance: 0.01<br>- CCs tolerance (%): 5%

**15**	**Spectral library search**<br>- Scans for matching: MS2 level >= 2 (merged)<br>- Precursor m/z tolerance: 0.001 m/z or 15 ppm<br>- Spectral m/z tolerance 0.001 m/z or 20 ppm<br>- Minimum matched signal: 4<br>- Weighted cosine similarity

Section S6: SIRIUS parameters

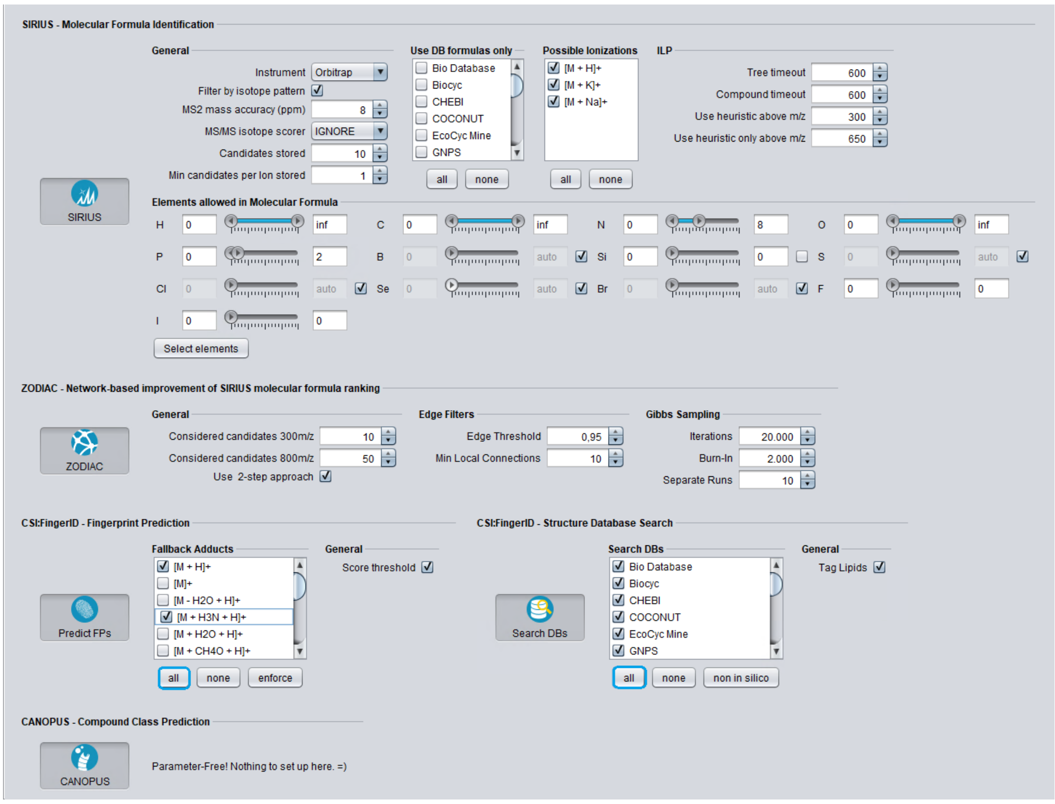

Figure S4. *SIRIUS parameters – ESI+.*

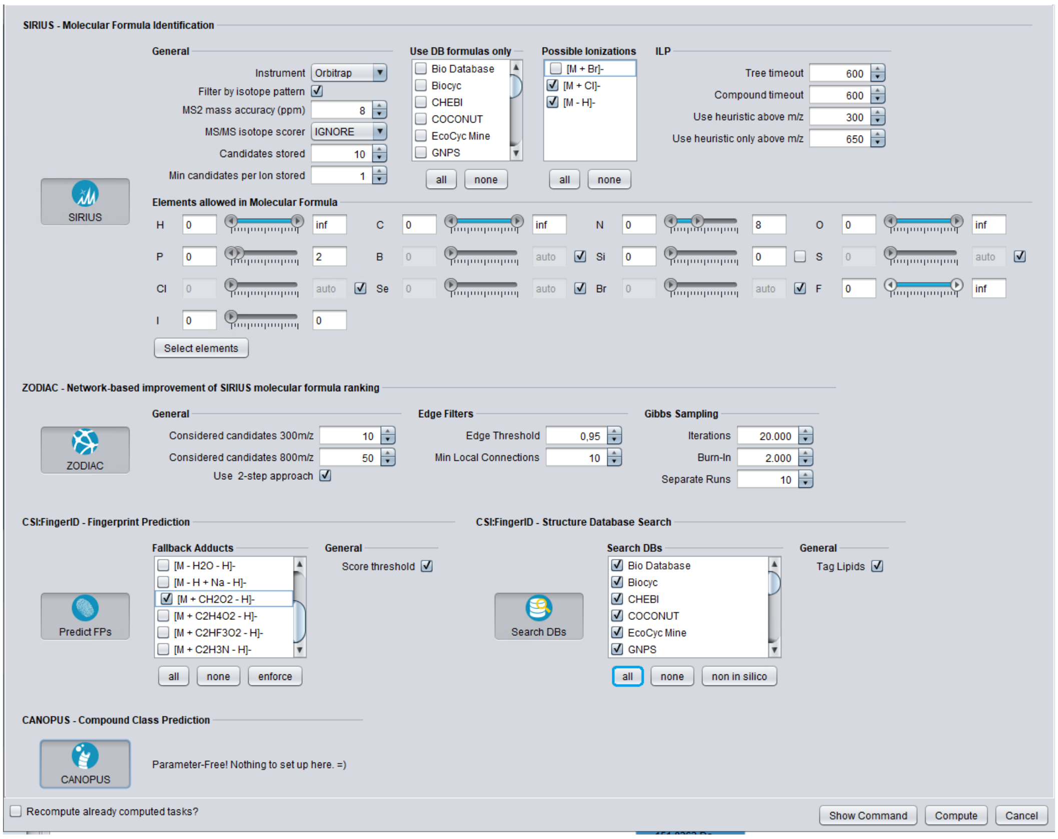

Figure S5. *SIRIUS parameters – ESI-.*

Section S7: Data pretreatment and cluster analysis results

To effectively derive temporal patterns and reduce biases from missing data or false negatives (e.g., by measurement errors or data processing techniques), only target compounds with a detection frequency over 85% were used for the cluster analysis, reducing the target list from 402 targets to 169 (Figure S6 A). Aiming to differentiate actual patterns from deviations appearing through the measurement, targets were prioritized based on variance, indicated by the relative standard deviation (RSD), which was compared against the RSD of internal standards. Only compounds with an RSD at least 1.5 times higher than the highest internal standard RSD (30%) were retained, resulting in 95 target compounds with variances exceeding 45% for further cluster analysis (Figure S6 B).

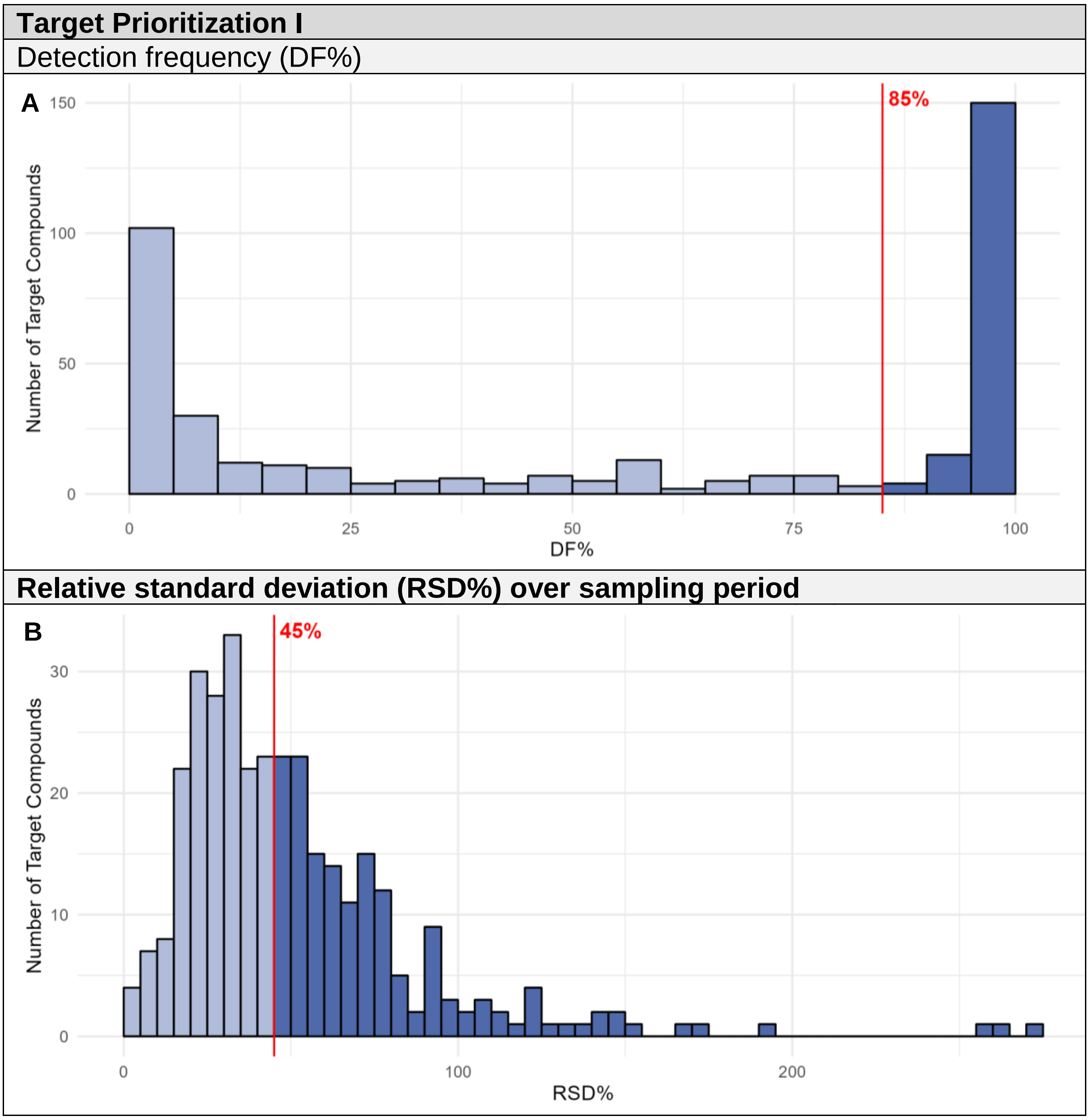

Figure S6. *Cut-off value applied for (**A**) detection frequency and (**B**) relative standard deviation of target compounds in wastewater samples. The dark blue columns represent the retained data, the light blue columns indicate the removed data.*

Exploring different data treatment strategies for the cluster analysis revealed similar patterns. However, a clustering with each day individually presented challenges in comparing clusters across days due to different results for day 1 due to the rain event. The rain event, which occurred on the night of the first day of sampling, led to weather-specific behavior that complicated the comparison to the other two days (Figure S7). On the other hand, averaging the data across all days obscured these weather-induced variations, resulting in a loss of valuable insights (Figure S8).

Approach 1: Individual clustering of each sampling day

Day 1

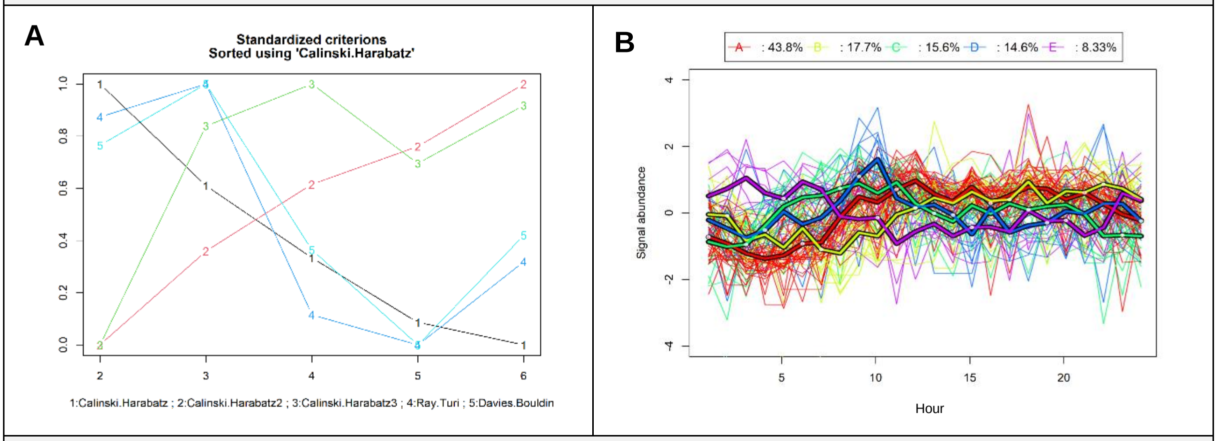

Day 2

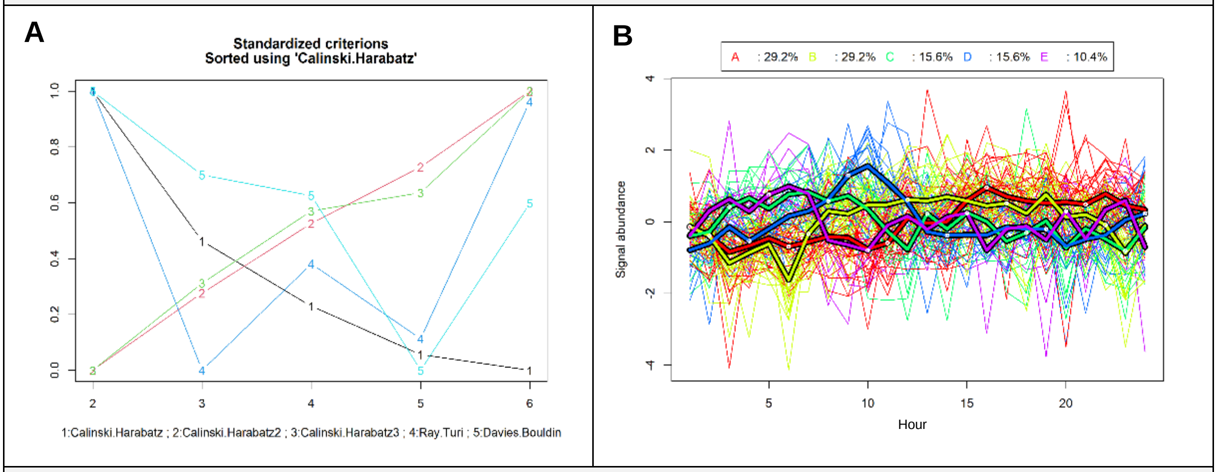

Day 3

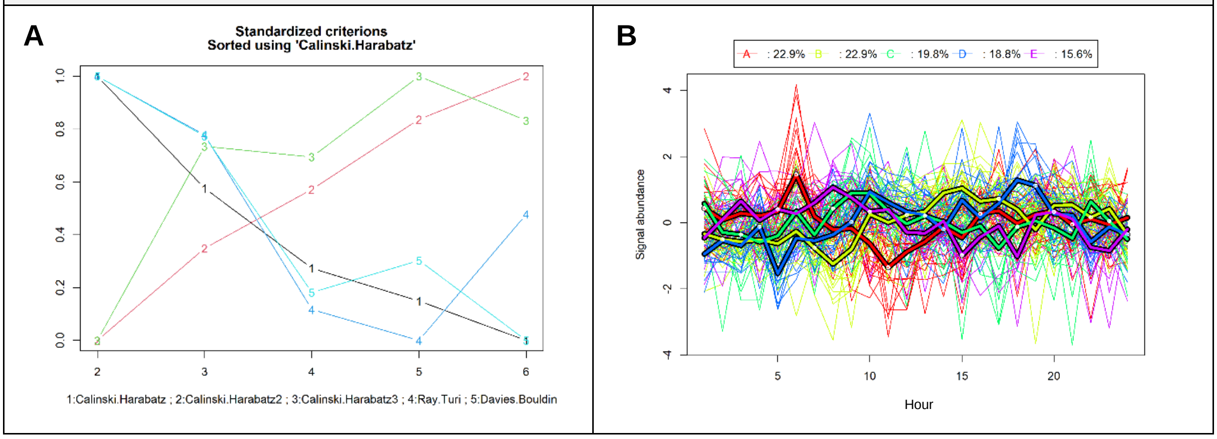

Figure S7. *Quality criteria (**A**) & cluster pattern (**B**) when clustering each sampling day individually. For each day between three and six clusters were suggested (number with most quality criteria maximized), but five clusters were plotted to enable comparison. The first day shows different patterns with an increased impact of the flow rate and cluster E showing a pattern that could not be observed on any other day.*

Approach 2: Averaging of all three sampling days

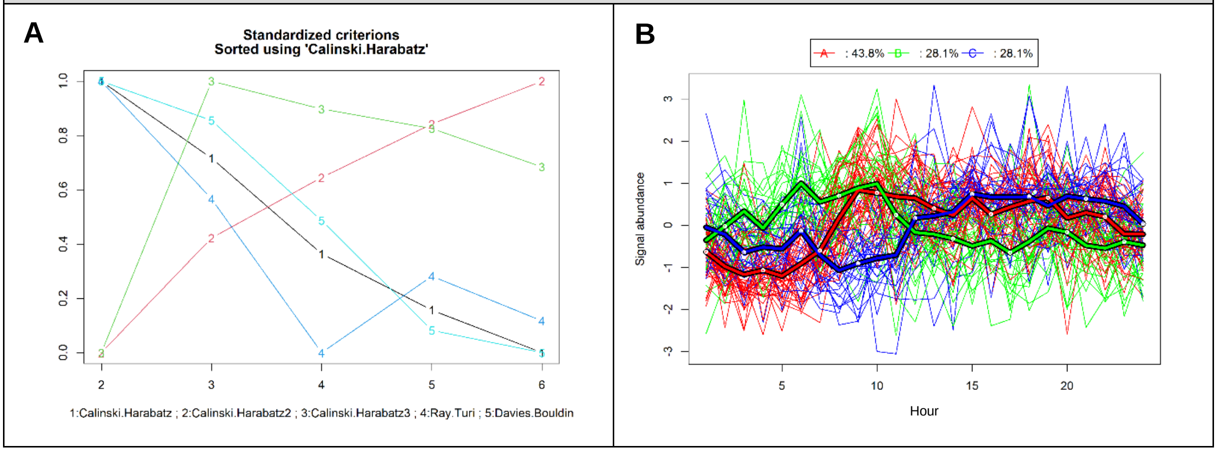

Figure S8. *Quality criteria (**A**) & cluster pattern (**B**) when clustering the data as average of each data point over the day (n =24) for all three sampling days. Three clusters were suggested, but the effect of the rain event was not observable anymore.*

Consequently, data was treated as a continuous 72-hour dataset, allowing clustering based on recurring patterns across all days while still observing the impacts of weather variations, particularly the rain event (Figure S9). The final number of clusters was chosen based on the quality criteria from the '*kml*' package, which suggested either three or five clusters for this dataset with the final approach (72-hours consecutive and no flow-rate correction). Both options were considered; however, interesting patterns among the five clusters, particularly during the rain event, led to the decision to choose this option. To address the challenge that every target compound is assigned to a cluster, regardless of the fit, targets were filtered based on their cluster assignment probabilities provided by the '*kml*' package. Compounds with probabilities below 90% were removed and only those with a good fit were kept for further interpretation (Figure S9 C).

Approach 3: 72-hour consecutive data set

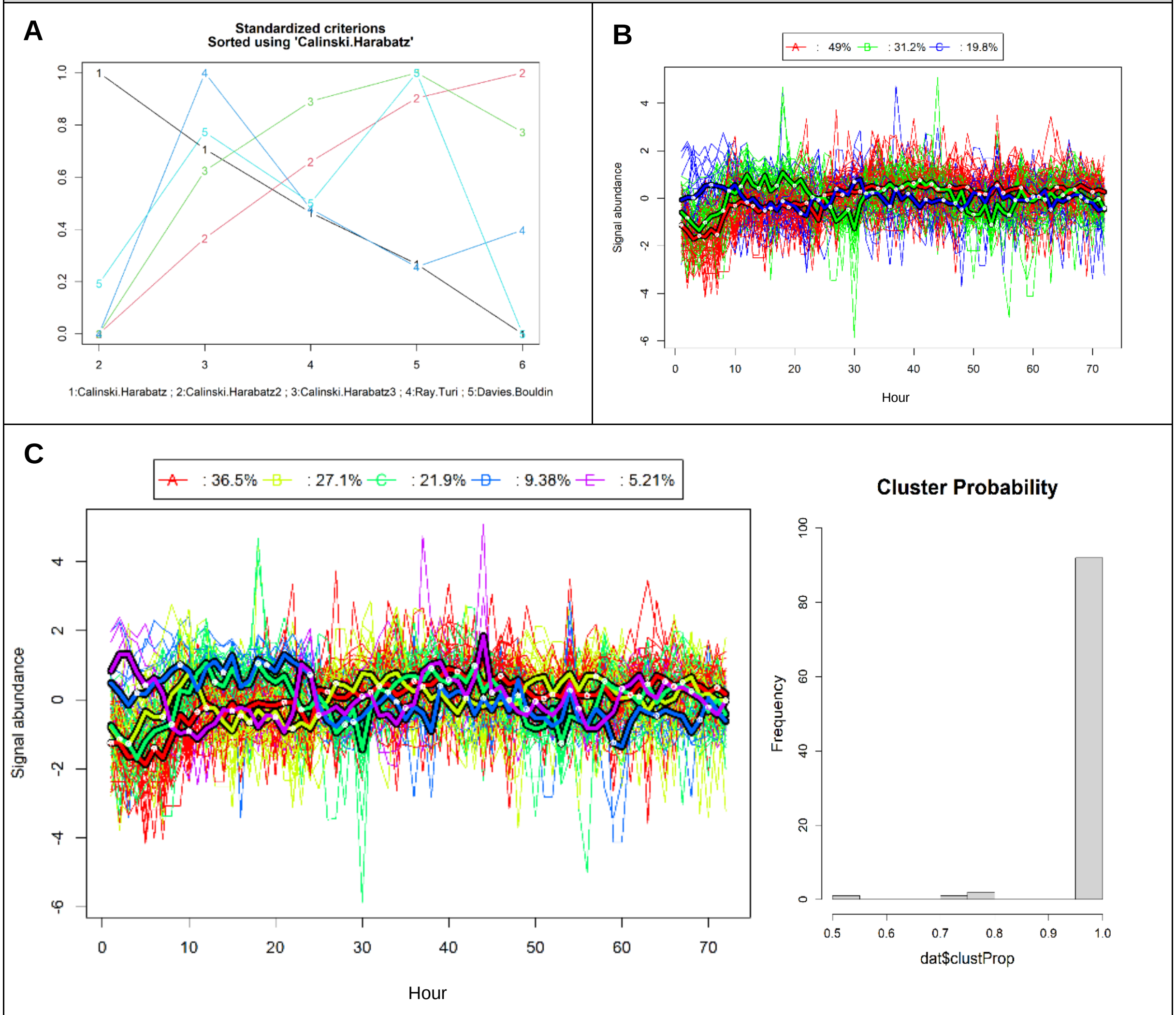

A = BG1, B = MP, C = DA, D = BG2, E = BG3

Figure S9. *Quality criteria (**A**) & cluster patterns if three clusters are extracted (**B**) vs. five clusters and the cluster probabilities (**C**) when clustering the data as a consecutive data set. Both scenarios (3 vs. 5 clusters), show the impact of the rain event, although with five clusters the result was more differentiated.*

The robustness of the temporal pattern between the sample days was evaluated by calculating the RSD of the target compounds, with 75% of the data (third quartile) showing an RSD below 50% (Figure S10).

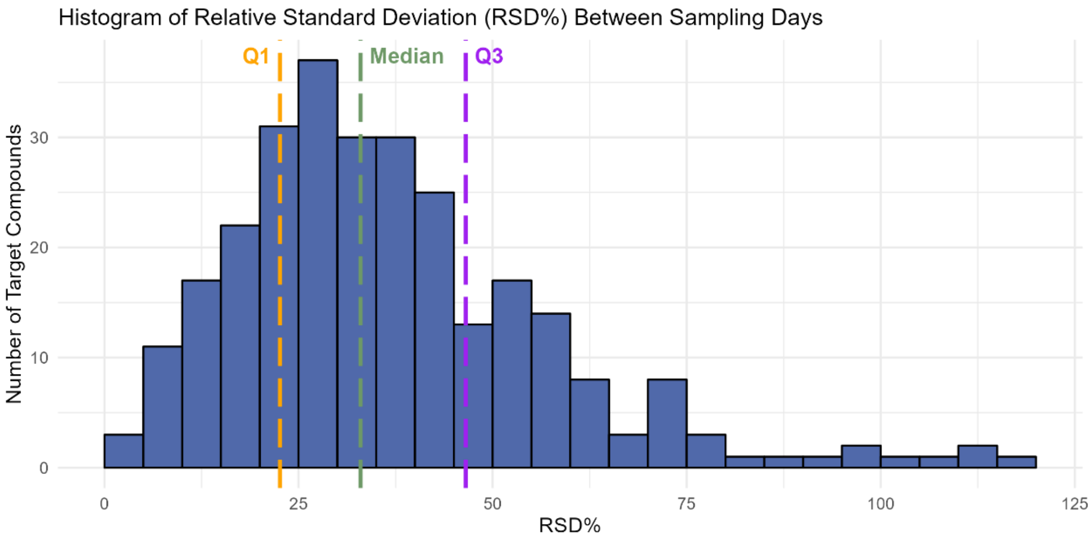

Figure S10. *Variability of signal intensities between sampling days.*

In wastewater analysis, measured concentrations are often normalized by the flow rate of the WWTP to obtain mass loads.[3–5] Normalizing signal intensities by flow rate before cluster analysis revealed similar trends to those without normalization but tended to overshadow the temporal patterns of some target compounds (Figure S11). Hence, we tended for the interpretation without normalization.

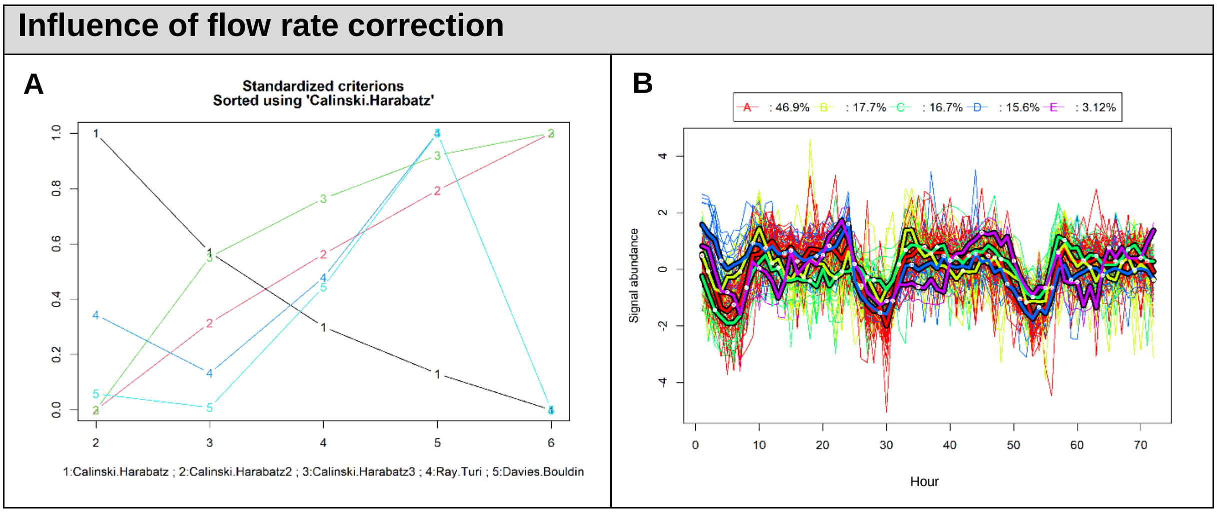

Figure S11. *Quality criteria (**A**) & cluster patterns (**B**) with flow rate correction, using the data as a consecutive data set. Five clusters were suggested and we could observe similar temporal pattern as in the main approach, also revealing a morning peak and day time clusters with later and longer maximum intensities.*

Performing the clustering on the samples in a randomized order confirmed that the cluster analysis yields meaningful results only when temporal trends are present (Figure S12).

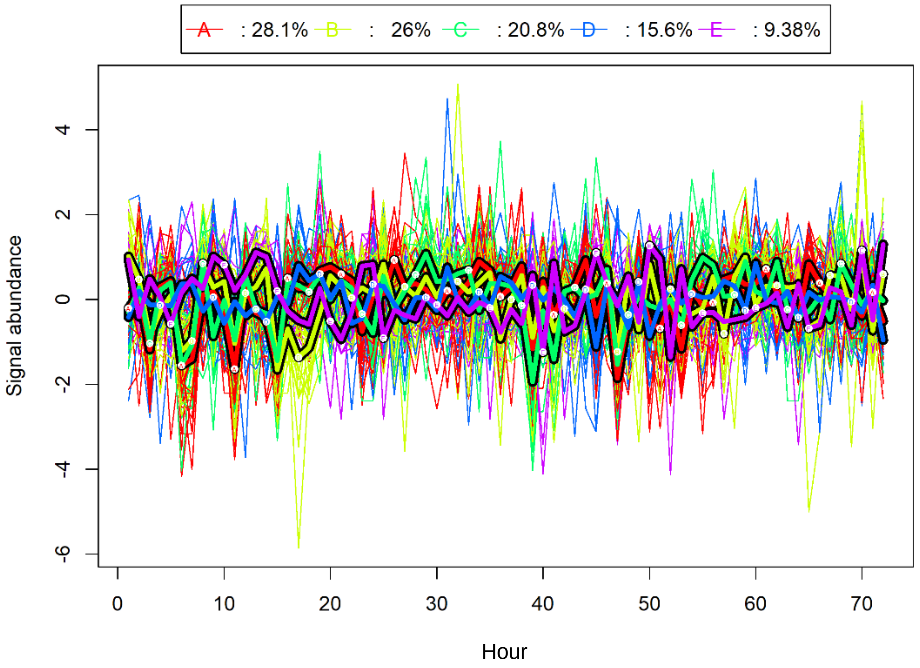

Figure S12. *Controlling the observed clustering as non-artefacts by randomized ordering of the sample days. Here, no patterns can be observed.*

Lastly, clustering was also performed on the complete non-target feature list to compare the results with those based on the target compounds. Even though similar patterns could be observed in both scenarios, the abundance on detected features made meaningful interpretations and feasible data processing steps challenging (Figure S13). Hence, the decision to focus on the target compounds for the first clustering.

Clustering results for all non-targeted features

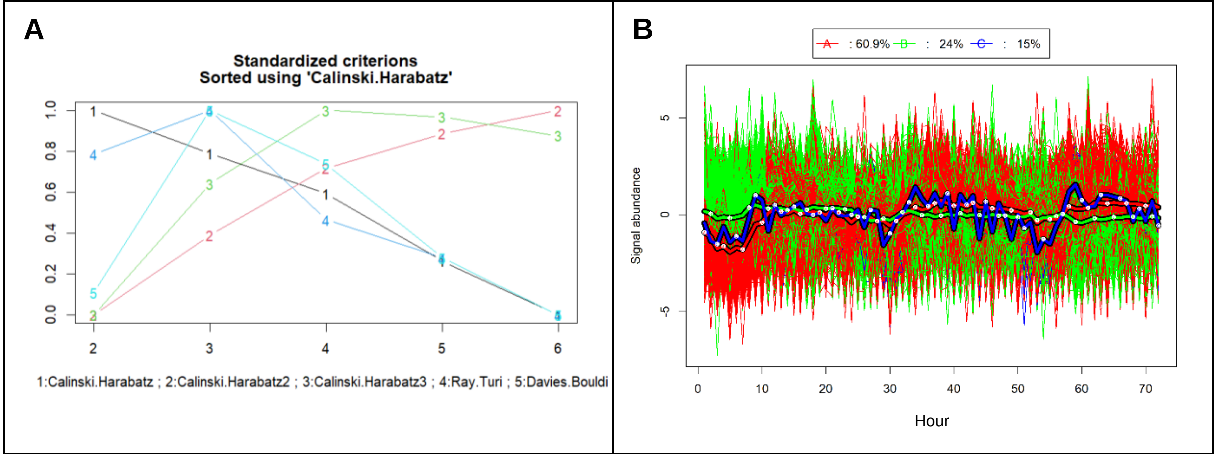

Figure S13. *Quality criteria (**A**) & cluster patterns (**B**) for a clustering of the non-targeted feature list. Here also, the main pattern of MP and DA cluster remain (labelled as cluster A and C).*

After extracting the cluster centroids of the selected cluster analysis (Figure S9 C), their correlation to the flow rate was assessed to understand how the observed clusters relate to flow rate fluctuations (Table S5). While clusters DA and BG2 show a positive correlation to the flow rate, clusters MP and BG1 show a negative correlation and BG3 shows no correlation.

Table S5. *Spearman rank correlation coefficients of all 5 clusters against the wastewater flowrate.*

Cluster	Spearman Correlation Coefficient (r_s)	p-value	Adjusted p-value
MP	-0.424	< 0.01	< 0.01
DA	0.428	< 0.01	< 0.01
BG1	-0.245	0.04	0.08
BG2	0.652	< 0.01	< 0.01
BG3	-0.078	0.516	0.516

Generalized additive models (GAMs) were used to emphasize overall trends and reduce noise from minor fluctuations in the cluster analysis output. The signal intensities of assigned targets were min-max normalized over 72 hours to facilitate pattern comparison rather than absolute intensities across compounds. To ensure no false connectivity was implied between sampling days, all targets in a specific cluster were used to fit individual GAMs for each of the three individual sampling days (Figure S14).

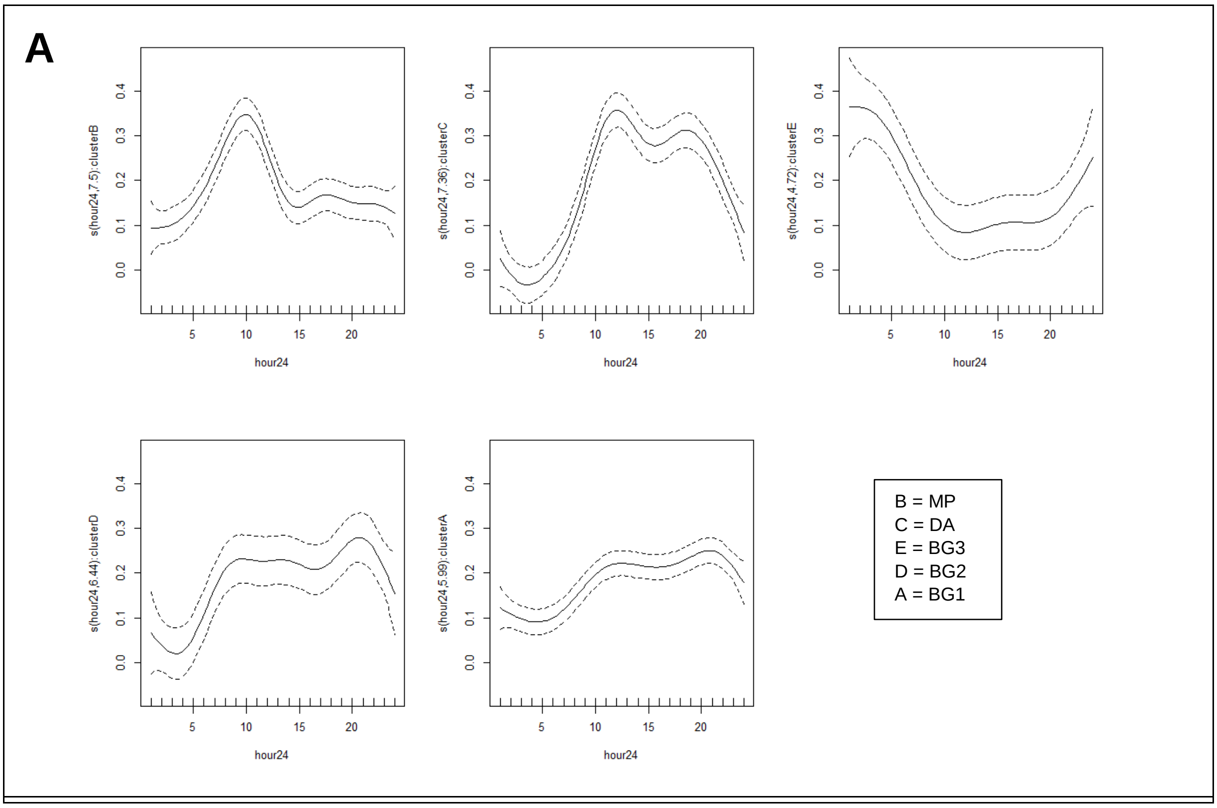

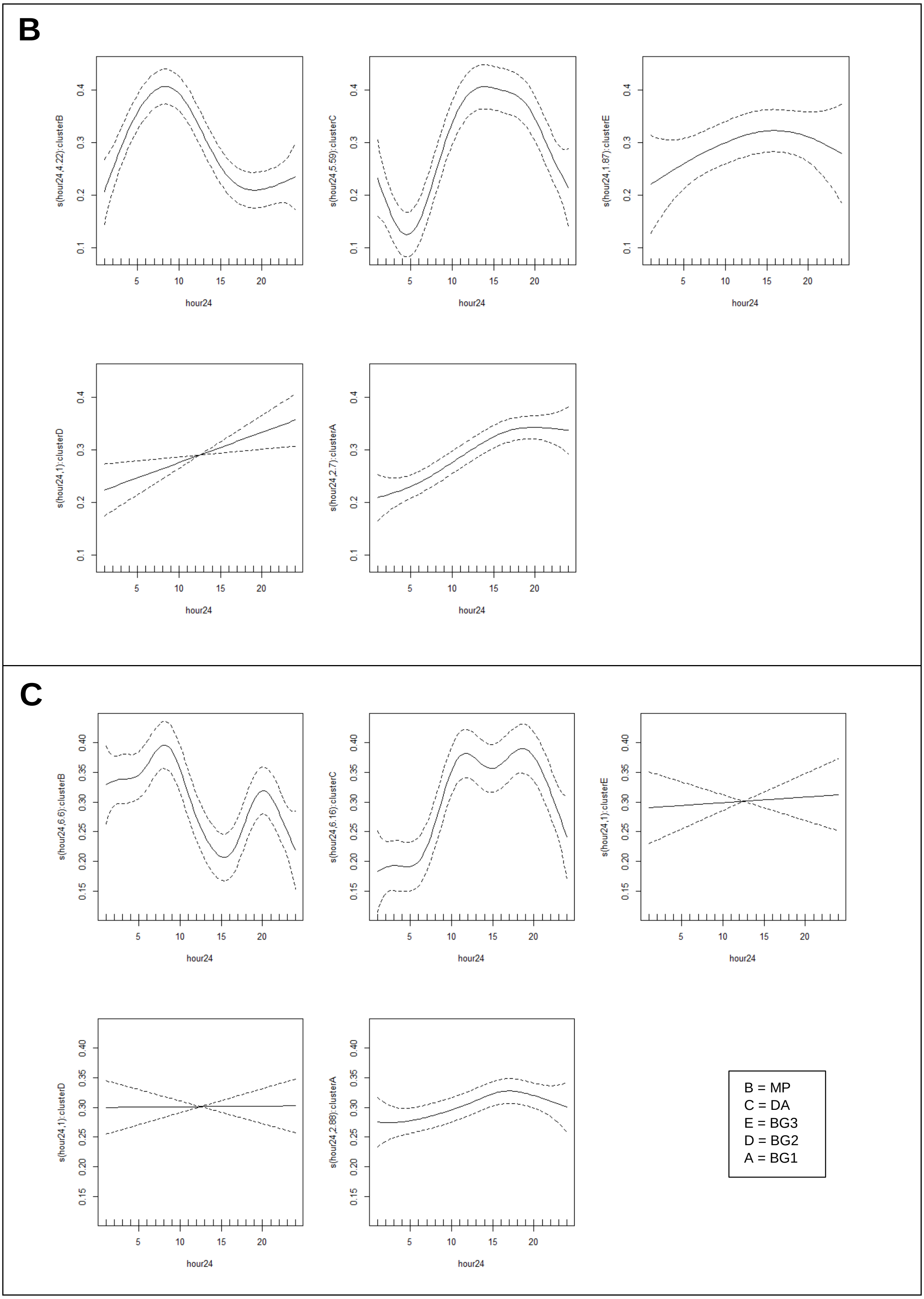

Figure S14. *Generalized Additive Model (GAM) calculated for the 5 cluster of (**A**) day 1, (**B**) day 2 and (**C**) day 3. Clusters A (BG1), D (BG2) and E (BG3) show a distinct pattern only on day 1.*

Section S8: Non-targeted feature prioritization

Feature Prioritization I

Detection frequency (DF%)

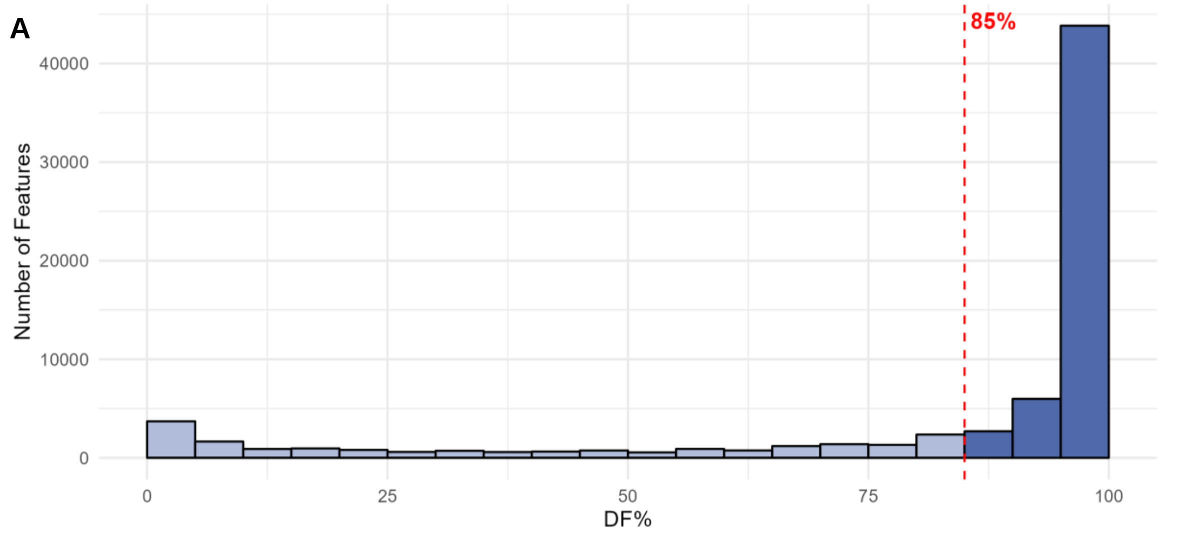

Relative standard deviation (RSD%)

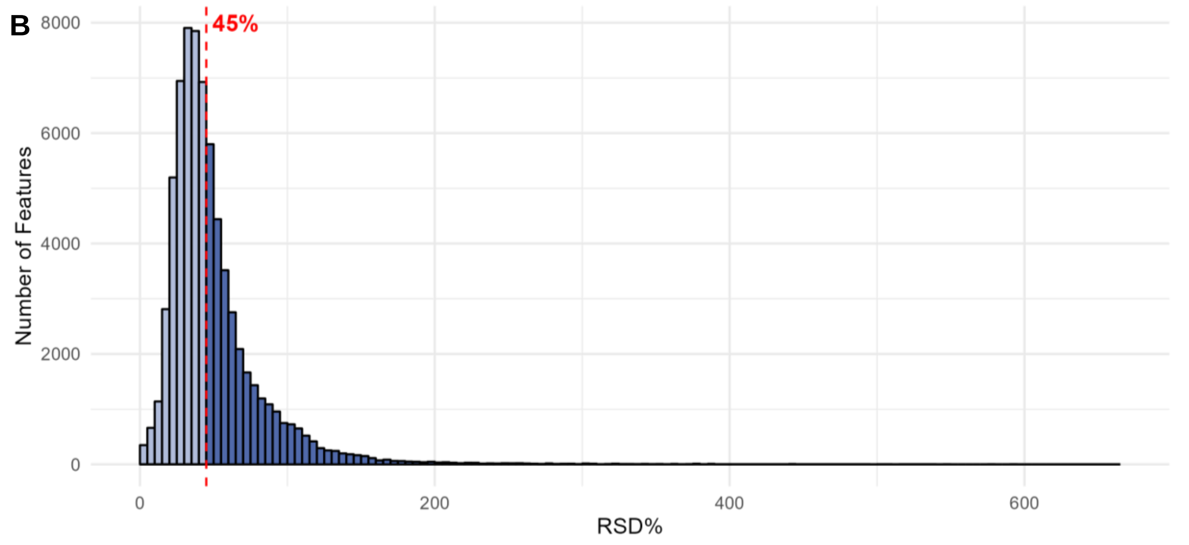

Figure S15. *Cut-off values applied for (**A**) detection frequency and (**B**) relative standard deviation on the selection of non-targeted features over the sampling period. The dark blue columns represent the retained data, the light blue columns indicate the removed data.*

Feature Prioritization II

Threshold 1: Distance ratio (closest to 2nd closest cluster)

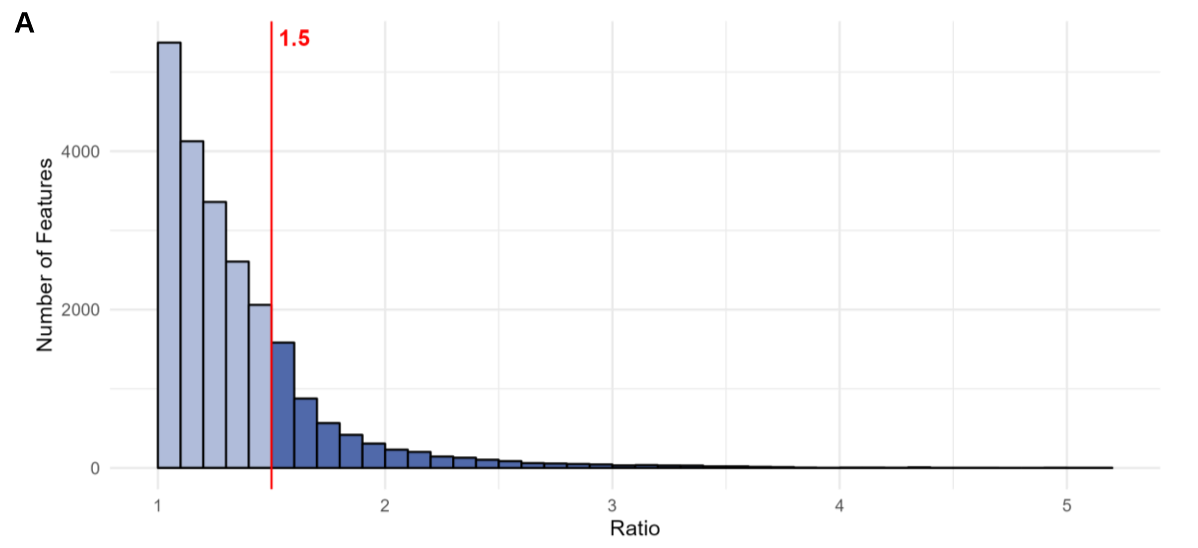

Threshold 2: Median of target compounds

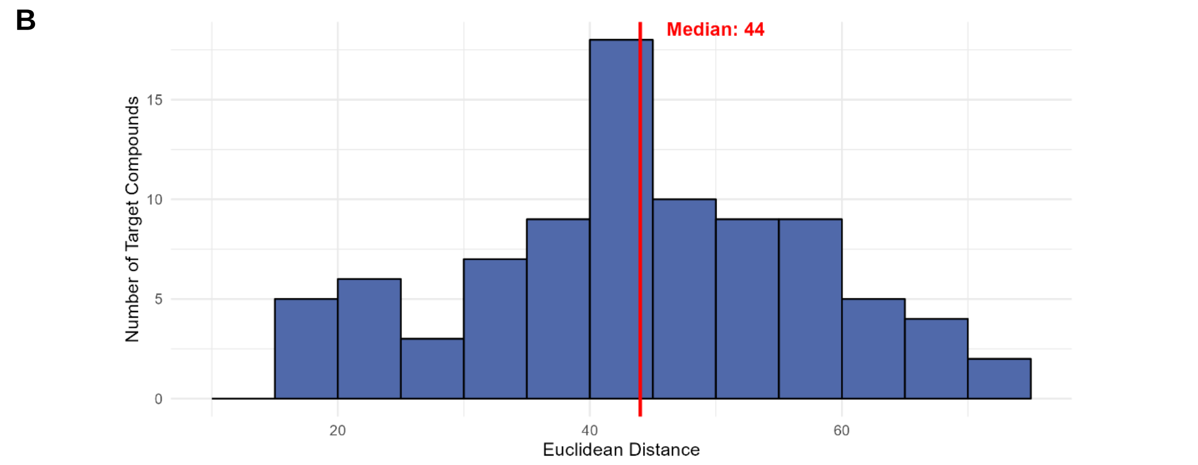

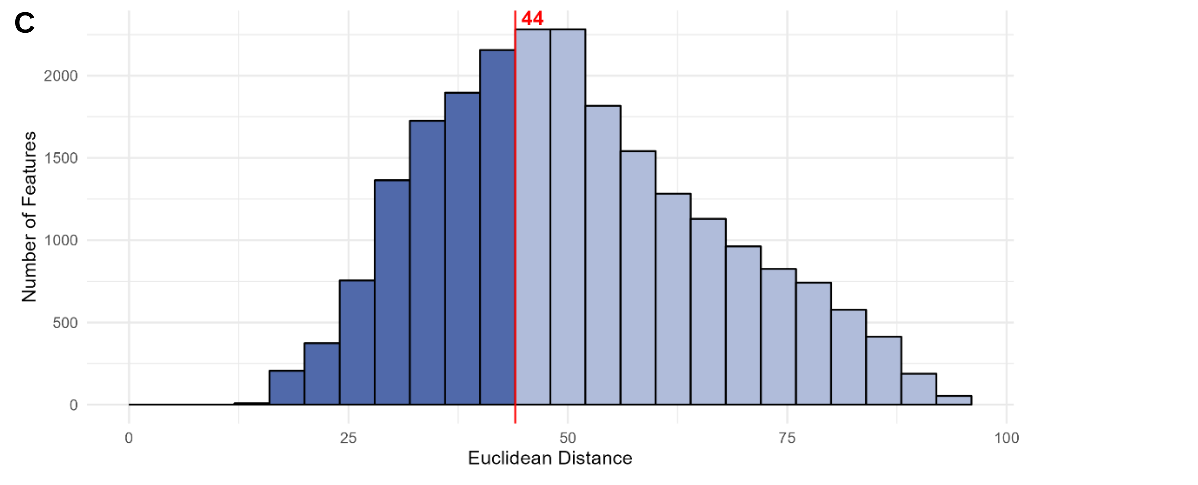

Figure S16. *Cut-off values for the non-targeted features based on distance ratio (closest vs. 2nd closest cluster) (**A**) and distance threshold (**C**) based on median distances observed for the target compounds (**B**). The dark blue columns represent the retained data, the light blue columns indicate the removed data.*

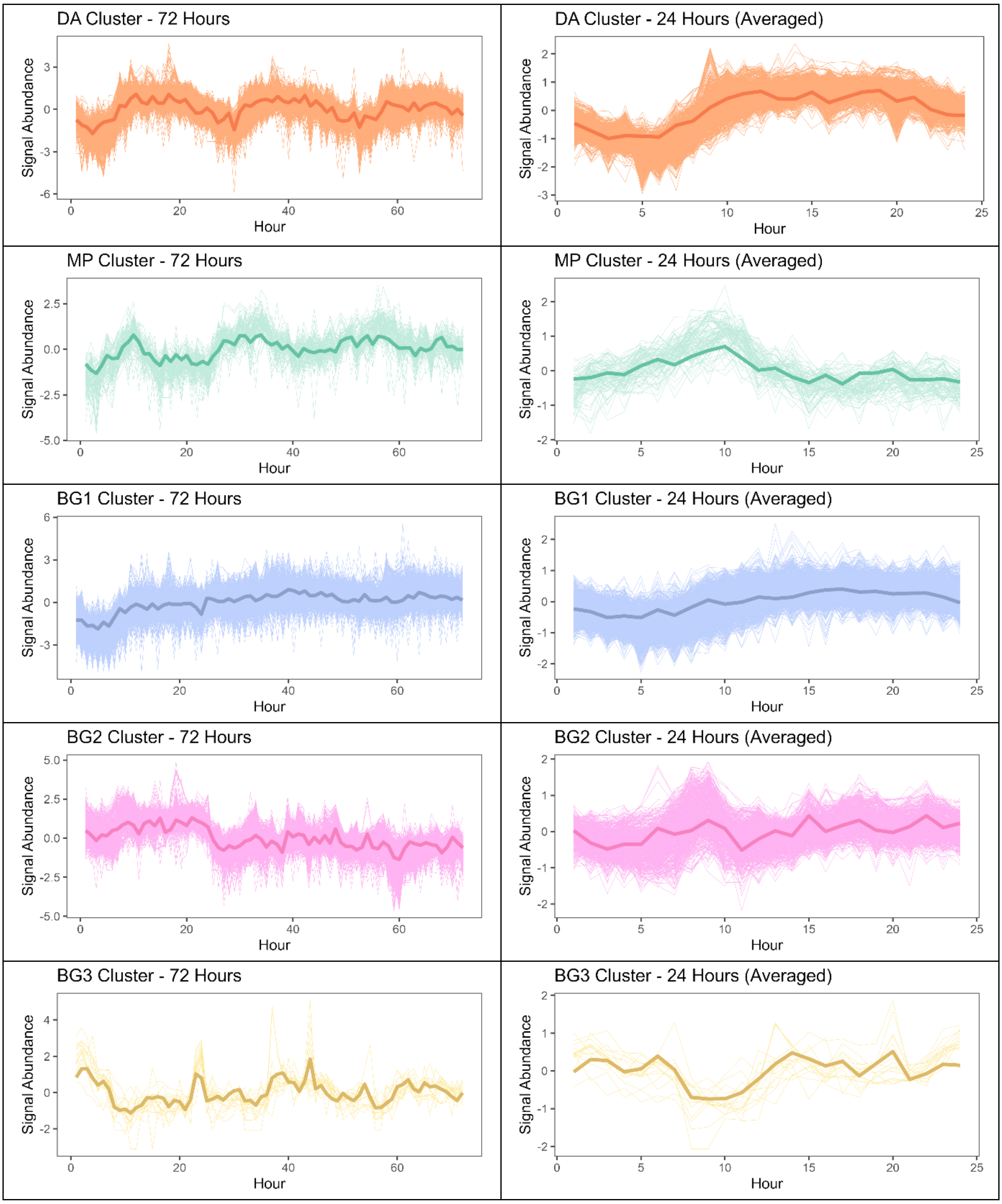

Figure S17. *Assigned non-targeted features for each cluster over 72 hours (left) and averaged over 24 hours (right). The cluster centroids are plotted as dark line in each graph.*

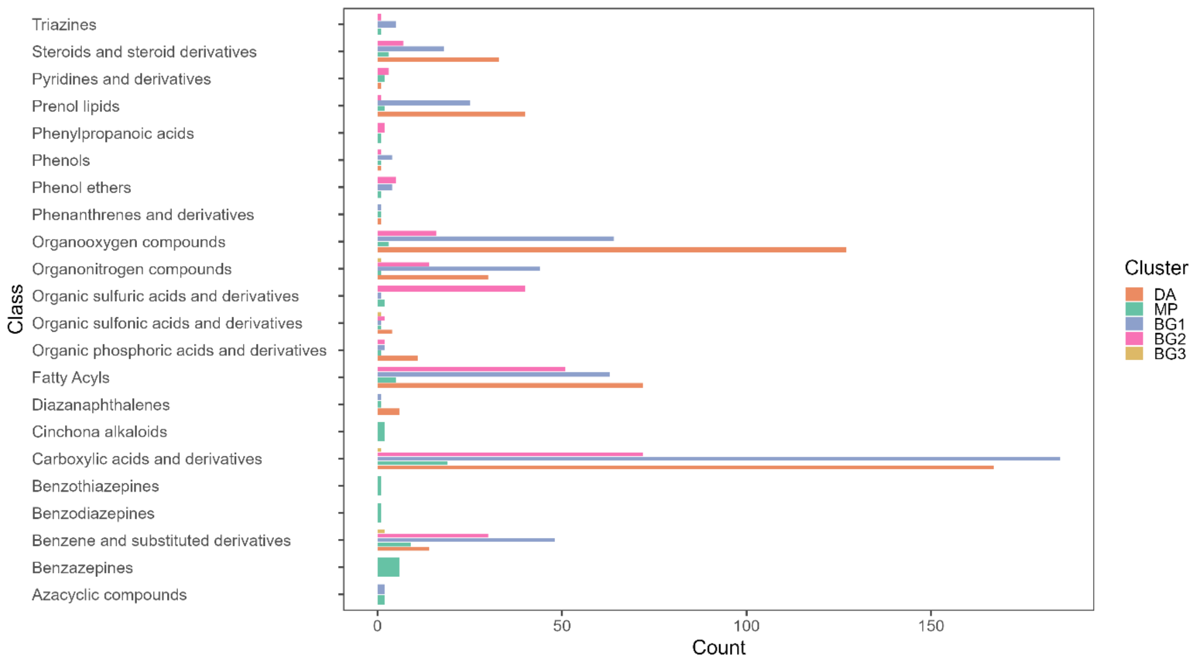

Figure S18. *ClassyFire Classes annotated in the non-target features of the morning peak cluster compared over all other clusters.*

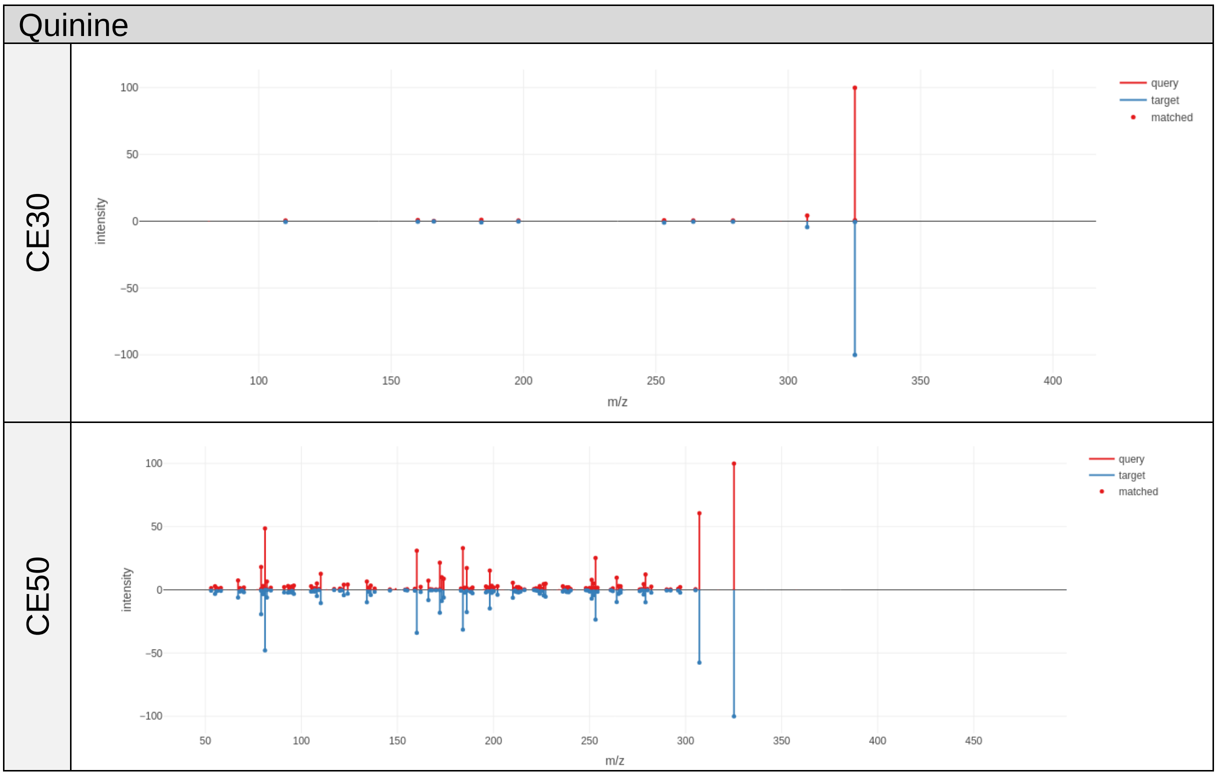

Figure S19.*Spectra comparison of the measured spectra in wastewater (query) and the reference spectra (target) for quinine in positive mode with an applied collision energy (CE) 30 and 50.*

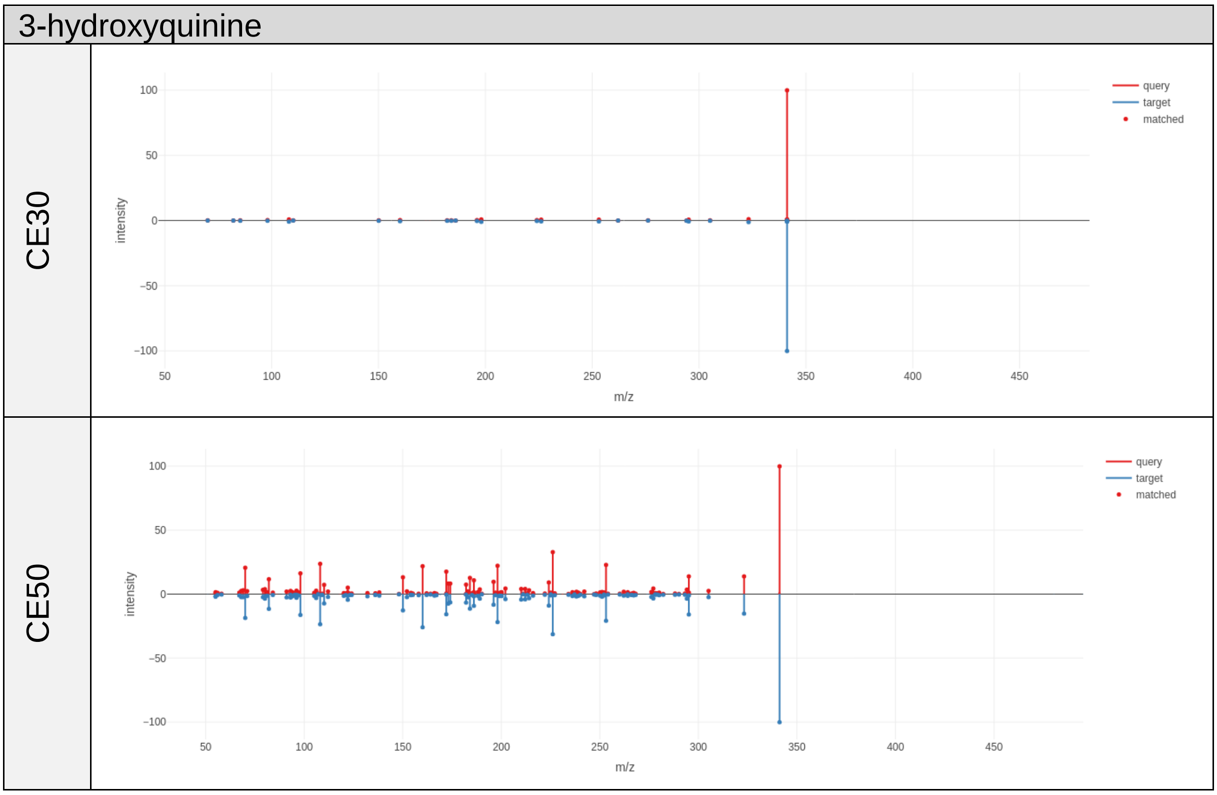

Figure S20. *Spectra comparison of the measured spectra in wastewater (query) and the reference spectra (target) for 3-Hydroxyquinine in positive mode with an applied collision energy (CE) 30 and 50.*

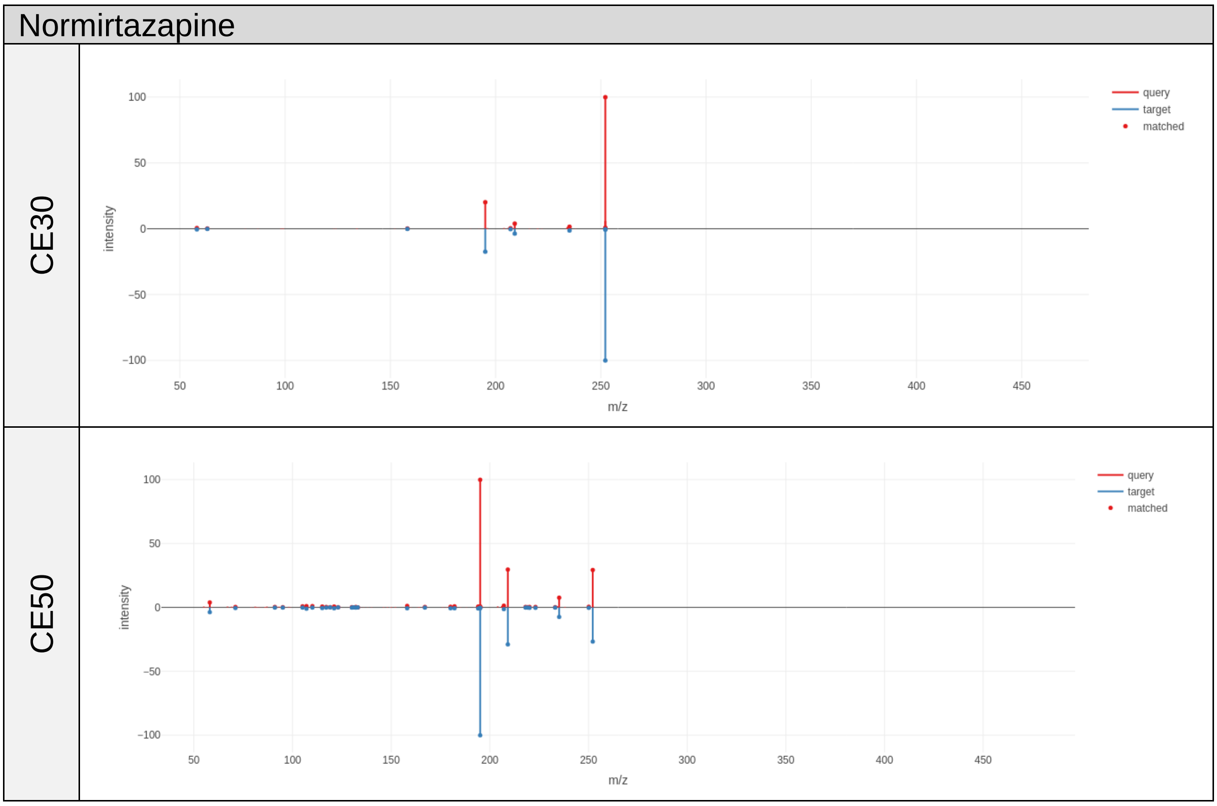

Figure S21. *Spectra comparison of the measured spectra in wastewater (query) and the reference spectra (target) for normirtazapine in positive mode with an applied collision energy (CE) 30 and 50.*

Urapidil

CE30

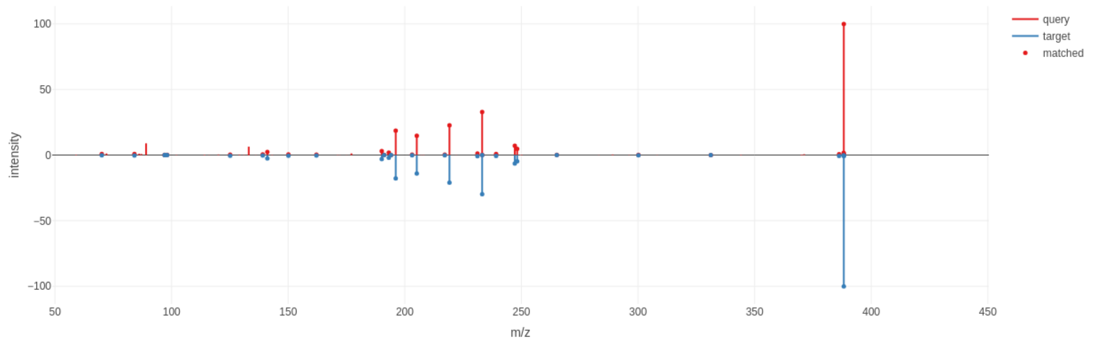

CE50

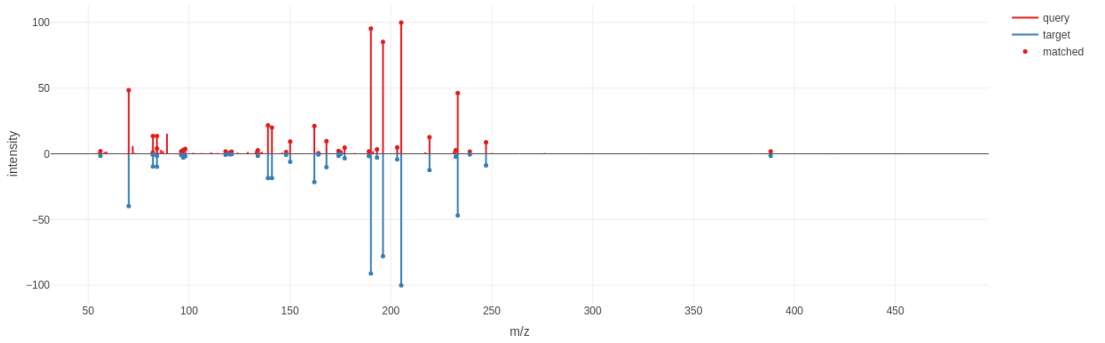

Figure S22. *Spectra comparison of the measured spectra in wastewater (query) and the reference spectra (target) for urapidil in positive mode with an applied collision energy (CE) 30 and 50.*

References

[1] Lopardo, L., Cummins, A., Rydevik, A., Kasprzyk-Hordern, B. (2017). New Analytical Framework for Verification of Biomarkers of Exposure to Chemicals Combining Human Biomonitoring and Water Fingerprinting. *Analytical chemistry*. 13, 7232–7239. DOI: 10.1021/acs.analchem.7b01527.

[2] Huber, C., Brack, W., Röder, S., Bergen, M. von, Rolle-Kampczyk, U., Zenclussen, A.C. et al. (2024). Pesticide residues and polyphenols in urine - A combined LC-HRMS screening to reveal intake patterns. *Environment international*. 108981108981. DOI: 10.1016/j.envint.2024.108981.

[3] European Union Drugs Agency (EMCDDA) (2016). Assessing illicit drugs in wastewater - Advances in wastewater-based drug epidemiology. Publications Office. ISBN: 9789291688562.

[4] Thomas, K.V., Bijlsma, L., Castiglioni, S., Covaci, A., Emke, E., Grabic, R. et al. (2012). Comparing illicit drug use in 19 European cities through sewage analysis. *The Science of the total environment*. 432–439. DOI: 10.1016/j.scitotenv.2012.06.069.

[5] Castiglioni, S., Bijlsma, L., Covaci, A., Emke, E., Hernández, F., Reid, M. et al. (2013). Evaluation of uncertainties associated with the determination of community drug use through the measurement of sewage drug biomarkers. *Environmental science & technology*. 3, 1452–1460. DOI: 10.1021/es302722f.
